# Neutrophil-initiated nociceptive ingrowth orchestrates inflammation resolution to potentiate bone regeneration

**DOI:** 10.1038/s41413-025-00481-6

**Published:** 2026-01-19

**Authors:** Xuanyu Qi, Guangzheng Yang, Zeqian Xu, Mingliang Zhou, Tejing Liu, Jiahui Du, Sihan Lin, Xinquan Jiang

**Affiliations:** 1 Department of Prosthodontics, Shanghai Ninth People’s Hospital, Shanghai Jiao Tong University School of Medicine, Shanghai, China; 2 College of Stomatology, Shanghai Jiao Tong University, Shanghai, China; 3 National Center for Stomatology, Shanghai, China; 4 National Clinical Research Center for Oral Diseases, Shanghai, China; 5 Shanghai Key Laboratory of Stomatology, Shanghai, China; 6 Shanghai Research Institute of Stomatology, Shanghai, China; 7 Shanghai Engineering Research Center of Advanced Dental Technology and Materials, Shanghai, China; 8 Shanghai Stomatological Hospital, Fudan University, Shanghai, China; 9 Department of Oral Pathology, Affiliated Hospital of Stomatology, Nanjing Medical University, Nanjing, China

**Keywords:** Bone, Diabetes complications

## Abstract

Nociceptive pain is a cardinal feature of traumatic and inflammatory bone diseases. However, whether and how nociceptors actively regulate the immune response during bone regeneration remains unclear. Here, we found that neutrophil-triggered nociceptive ingrowth functioned as negative feedback regulation to inflammation during bone healing. A unique Il4ra^+^Ccl2^high^ neutrophil subset drove intense postinjury TRPV1^+^ nociceptive ingrowth, which in return dissipated inflammation by activating the production of pro-resolving mediator lipoxin A4 (LXA4) in osteoblasts. Mechanistically, osteoblastic autophagy activated by nociceptor-derived calcitonin gene-related peptide (CGRP) suppressed the nuclear translocation of arachidonate 5-lipoxygenase (5-LOX) to favor the LXA4 biosynthesis. Moreover, in alveolar bone from patients with Type II diabetes, we found diminished nociceptive innervation correlated with reduced autophagy, increased inflammation, and impaired bone formation. Activating nociceptive nerves by spicy diet or topical administration of a clinical-approved TRPV1 agonist showed therapeutic benefits on alveolar bone healing in diabetic mice. These results reveal a critical neuroimmune interaction underlying the inflammation-regeneration balance during bone repairing and may lead to novel therapeutic strategies for inflammatory bone diseases.

## Introduction

Bone diseases are rapidly becoming a global health hazard. Generally, half of individuals over the age of 18 years live with bone diseases.^1^ Nociceptive pain, which originates from nociceptors and is sensitized by inflammatory mediators, is the hallmark of traumatic and inflammatory bone diseases.^2,3^ Yet nociceptive pain is an unpleasant sensation for most of patients, the reports on the negative impact of painkillers, such as nonsteroidal anti-inflammatory drugs (NSAIDs),^4,5^ on bone healing have led to the intriguing question of whether activating nociceptive nerves rather than suppressing them benefits bone healing. Previous studies have uncovered the importance of bone-innervating nerves in cell recruitment, angiogenesis, and osteogenic differentiation.^6–10^ However, healing process begins with an inflammatory reaction that decisively influences the subsequent phases^11^ the effects of nerves on this decisive early phase of bone regeneration remains unknown.

Recent studies have revealed the potential immunoregulatory role of nociceptive nerves in different organs.^12–15^ Studies have increasingly shown that nociceptors are not simply bystanders but rather regulators that dynamically interact with immune cells through cell-cell contacts^16,17^ or the release of neurotransmitters.^13,15^ However, the full picture of the relationship between nociceptive neurons and the immune system is far from complete. Depending on the context, nociceptive neurons can either exacerbate^17–19^ or suppress^12,13,20^ inflammation, impede host defense, or promote tissue repair. Bone is densely innervated by nociceptive nerves,^21,22^ but very little is known about the immunoregulatory role of nociceptors during bone regeneration. Here, we investigated the importance of neuroimmune interactions in bone healing and uncovered a previously unknown mechanism for the endogenous feedback control of inflammation by neutrophil-initiated nociceptive ingrowth.

## Results

### TRPV1^+^ nociceptive innervation resolves inflammation

**Fig. 1:**
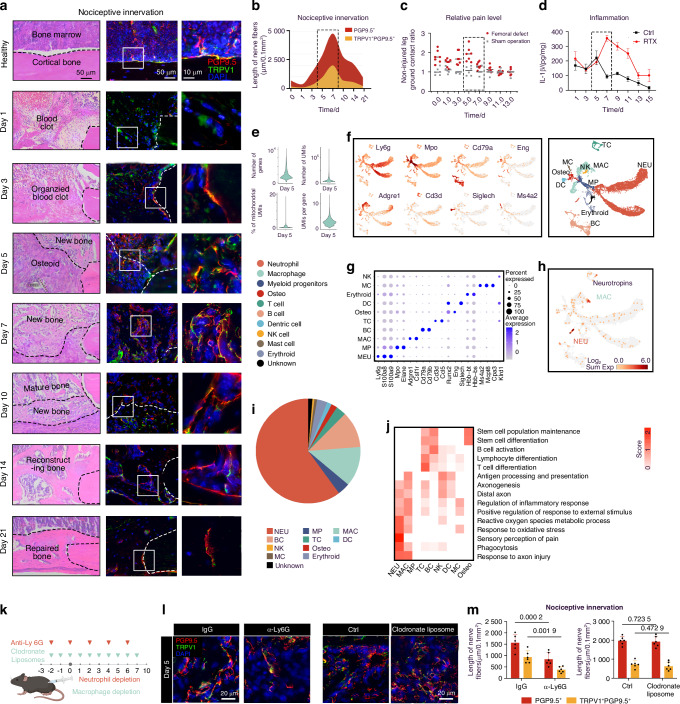
A negative feedback loop between neutrophils and nociceptors. **a** Representative images of H&E staining (left), immunostaining (middle) and high-magnification images (right) of the nociceptive nerves immunohistochemical stained for PGP9.5 (red)/ TRPV1 (green) at serial time points between days 1 and 21 after bone injury. scale bars: 50 μm or 10 μm. **b** The stream chart presents a semi-quantitative analysis of nerve fiber length, with the width of each stream representing the fluorescent area at different time points. **c** Relative pain level (non-injured/injured leg ground contact time ratio) in C57BL/6 mice at different time points post femoral injury or sham operation (*n* = 7 mice in femoral injury group, *n* = 6 mice in sham operation group). **d** IL-1β levels in femurs of Vehicle- or RTX-treated C57BL/6 mice at different time points post femoral injury (*n* = 6 femurs per group). **e** Violin plots of the number of genes, number of UMIs, mitochondria count percentage, and UMI per gene of all QC-passed cells in day 5. **f** Uniform manifold approximation and projection (UMAP) of 13 902 cells from the regenerating bone colored by cell type, Expression of unique genes specifically distinguished each cluster and associated them with neutrophil (NEU) (Ly6g, S100a8 and S100a9), myeloid progenitor (MP) (Mpo and Elane), macrophage (MAC) (Csf1r, Adgre1), B cell (BC)(Cd79a, Cd79b), T cells (T cell) (Cd3d, Ccl5), and dendritic cell (DC) (Siglech), osteolineage cell (Runx2, Eng), mast cell (MC) (Ms4a2, Mcpt8 and Cpa3), NK cell (NK) (Klrd1) and erythroid (Hbb-bt, Hbb-bs), respectively. **g** Scaled expression of selected signature genes for each cluster. **h** Expression of canonical markers of the main cell lineages and genes encoding canonical neurotrophins by color. **i** The proportion of each cell lineages from day 5. **j** Gene Ontology analysis of DEGs for each cell lineage. Selected Gene Ontology terms with *P* values < 0.05 (one-sided Fisher’s exact test) are shown and colored by relative score across each cell lineages. **k** Schematic diagram of depletion of neutrophils and macrophages. **l** Representative images and (**m**) semi-quantative analysis of nociceptive nerves immunostained for PGP9.5 (red)/ TRPV1 (green) on day 5 (*n* = 6 femurs per group). scale bars: 20 μm. Data are mean ± s.e.m. and are representative of at least four independent experiments. *P* values determined by two-way ANOVA

We first explored the distribution of TRPV1^+^ nociceptors during bone healing in a previously validated murine femoral diaphysis defect model.^23–26^ The healing of this defect model exhibited typically “inflammation-repair-remodel” timeline as previous described^27^ over a 21-day period. The inflammation-repair transition occurred on day 5 post injury, and new bone formation was observed on day 7 (Fig. 1a, Extended data Fig. 1a). Nociceptive fibers (TRPV1^+^ PGP9.5^+^) started to extend on day 3, reaching the peak on day 7 (Fig. 1a, b). Pain perception evaluated based on gait asymmetry,^28–30^ intensified as the ingrowth of nociceptors until day 5 (Fig. 1c). However, we serendipitously discovered a contradiction between them: pain responses in mice drastically declined on day 7, when nociceptive innervation was most evident. We deduced reduced inflammation might contribute to such alleviated pain behaviors. As expected, level of canonical inflammation mediator interleukine-1β (IL-1β) decreased substantially on day 7 (Fig.1d). Correlation analysis showed that the expression of *Gap43*, a marker of nerve regeneration,^31,32^ was negatively correlated with that of *Il-1β* on day 7 (Extended data Fig. 1b) but was positively correlated with those of anti-inflammatory gene *Il-10* and osteogenesis gene *Alp* (Extended data Fig. 1b). These results indicated a potential negative correlation between early-stage inflammation and nociceptive innervation. We further targeted ablated bone-innervating TRPV1^+^ neurons by injecting Resiniferatoxin (RTX) in L3-L5 DRGs (Extended data Fig. 1c, d). Nociceptive denervation led to abnormal increase in inflammatory cytokines (Extended data Fig. 1e) and mature neutrophil infiltration (Extended data Fig. 1f) in the injured sites, ultimately leading to impaired bone regeneration (Extended data Fig. 1g, h). Collectively, these results suggest that nociceptive innervation serves as a brake to restrain inflammation in early phase of bone healing.

### A negative feedback loop between neutrophils and nociceptors

Ablation of nociceptors resulted in aberrant infiltration of neutrophils (NEUs) rather than macrophages (MACs) or other immune cells on day 7 (Extended data Fig. 1f). Specifically, we observed significant accumulation of neutrophil extracellular traps (NETs) in the injured sites of RTX-treated mice (Extended data Fig. 2a, b). NETs have been proved to impair bone formation through potentiating the formation of osteoclasts, exaggerating the inflammatory response, and inducing the pro-inflammatory phenotype of macrophages.^33,34^ Degrading NETs by intraperitoneal injection of type I deoxyribonuclease (DNase I)^35^ partially mitigated impaired bone formation induced by RTX-treatment (Extended data Fig. 2c, d), indicating that inhibition of bone repair following nociceptive denervation was, at least in part, due to increased neutrophilic influx.^33,36^ Intriguingly, although the number of NEUs decreased concordantly with the most active nociceptive ingrowth on day 7, the NEUs infiltrated the injured sties from postoperative days 1 to 5, coinciding with the initial nerve ingrowth (Extended data Fig. 2e). The pain level was also positively correlated with the fluorescent area of NEU marker Ly6G (Extended data Fig. 2e, f), rather than that of MAC—which have been previously reported to promote nerve sprouting.^9,37^ Moreover, Spearman analysis also revealed a positive correlation between the expression of neutrophil chemotaxis marker gene *Cxcr2*^38^ and that of axon sprouting marker gene GAP43^32^ within 5 days after injury (Extended data Fig. 2g). Therefore, we hypothesized that nociceptive ingrowth might serve as negative feedback on neutrophilic influx.

To test the hypothesis, we performed single-cell RNA sequencing to get a deeper insight into such neuro-immune interaction (Fig. 1e–j). 10 major cell populations were identified in the injured sites on day 5 (Fig. 1f, g). UMAP analysis indicated that genes encoding canonical neurotrophins were mainly expressed in neutrophils, with a small portion in macrophages (Fig. 1h), while genes involved in axonogenesis, especially in sensory perception of pain mainly enriched in neutrophils according to Gene Ontology analysis of different expression genes (DEGs) (Fig. 1j). To verify the contribution of these two kinds of cells to nociceptive sprouting, we depleted neutrophils (NEUs) and macrophages (MAC) with Ly6G neutralizing antibodies (αLy6G)^39^ and clodronate liposomes (CL),^40^ respectively (Fig. 1k). Depleting either of them did not significantly change the number of the other in injured sites on day 5 (Extended data Fig. 2i, j, m), yet depletion of NEUs, instead of MACs, significantly inhibited the ingrowth of both PGP9.5^+^ nerve fibers and TRPV1^+^PGP9.5^+^ nociceptive nerve fibers (Fig. 1l, m). Considering the prolonged presence of MACs during bone healing (Extended data Fig. 2e), the observation of nociceptive ingrowth in clodronate liposome-treated mice was continued until day 10. In both the control group and the CL group, the number of nociceptors decreased after 7 days. However, in the CL group, macrophage clearance led to increased neutrophil infiltration after 7 days, while the rate of nociceptor decrease was significantly alleviated, suggesting the minor influence of MACs but critical role of NEUs in nociceptive ingrowth during early phase (before day 5). (Extended data Fig. 2k–n), suggesting the minor influence of MACs in early phase nociceptive ingrowth. Collectively, these results depicted a negative feedback loop between neutrophils and nociceptors during early stage (before day 5) of bone healing: neutrophils initiate nociceptive nerve sprouting, which in return constrains the neutrophilic infiltration to trigger bone repair.

### Neutrophils initiate nociceptive ingrowth via NGF-TrkA axis

**Fig. 2:**
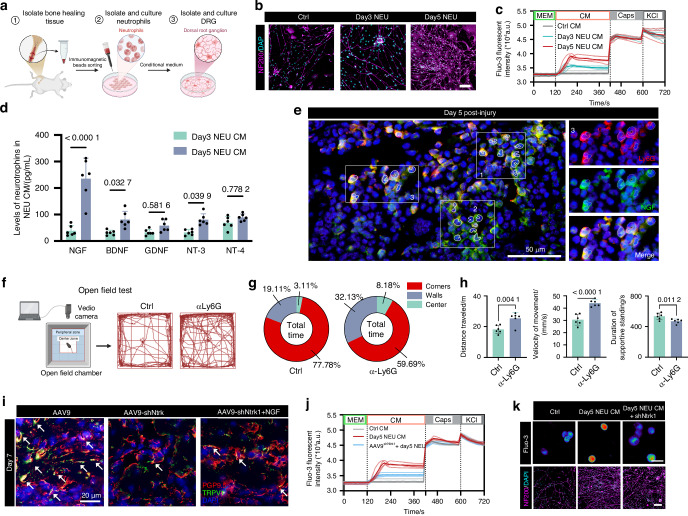
Neutrophils initiate nociceptive ingrowth via NGF-TrkA axis. **a** Schematic diagram illustrating the isolation of neutrophils and the conditioned culture of DRG. **b** Neurite outgrowth of DRG neurons treated with either normal medium or conditioned medium collected from neutrophils (NEU CM) isolated on day 3 or day 5 post bone injury. Scale bars, 50 μm. **c** Calcium influx in KCl-responsive wild-type DRG neurons triggered by NEU CM or capsaicin. **d** Levels of a panel neurotrophins in NEU CM (*n* = 6 biologically independent samples). **e** Representative images of immunostaining of NGF (Green) and LY6G^+^ neutrophils (Red). **f** Schematic diagram and representative images of open field test. **g** Time spent in the center and peripheral areas during the open field test. **h** Detailed movement analysis of mice treated with either murine IgG1 or α-Ly6G neutralizing antibody (*n* = 6 mice per group). **i** Representative images of TRPV1^+^ (green)/ PGP9.5^+^ (red) nociceptive nerve fibers in defects of femurs isolated on day 7 from TRPV1-cre mice with indicated treatments. Scale bars, 20 μm. **j**, **k** Calcium influx and neurite outgrowth in wild-type or Ntrk1 knockdown DRG neurons triggered by Day5 NEU CM. (*n* = 4 biologically independent samples). Scale bars, 50 μm. Data are mean ± s.e.m. and are representative of at least three independent experiments. *P* values determined by two-way ANOVA (**d**) and Student’s *t*-test (**h**)

To identify the potential mechanism underlying the neuroimmune interaction, we isolated neutrophils from injured sites on the 3rd and 5th days post-injury, respectively. The conditioned medium of neutrophils (NEU CM) was collected for DRG neuron culture (Fig. 2a). Compared to day 3 NEU CM, day 5 NEU CM resulted in more significant neurite outgrowth and Ca^2+^ influx in vitro (Fig. 2b, c). Boiling NEU CM lost its ability to promote neurite outgrowth, whereas pretreatment with DNase or RNase retained this ability (Extended data Fig. 3a). The results indicated that soluble proteins in NEU CM play a major role in promoting neurogenesis. Therefore, we performed a multiplexed enzyme-linked immunosorbent assay (ELISA) to screen NEU CM for a panel of neurotrophins and found that nerve growth factor (NGF) exhibited the most significant upregulation among five neurotrophins in day 5 NEU CM compared to day 3 NEU CM. (Fig. 2d). A cluster of Ly6G^+^NGF^+^ cells was confirmed in healing femurs on day 5 (Fig. 2e).

We further performed locomotor behavior analysis to evaluate nociceptor-mediated pain responses on day 5^41,42^ (Fig. 2f–h). Locomotor behaviors in mice treated with Ly6G antibodies was minimally affected by pain, as manifested by greater overall movement distances, increased movement speeds, and shorter durations of supportive standing after injury when compared to control group (Fig. 2g, h). Moreover, impaired bone healing in αLy6G group ruled out the potential influence of structural integrity of bone to locomotor activities (Extended data Fig. 3b, c). These results underscored again the importance of neutrophils in nociceptive nerve sprouting. As expected, neutrophil depletion also resulted in a significant decrease in the gene (Extended data Fig. 3d) and protein (Extended data Fig. 3e, f) level of *Ngf* in the injured sites.

Given that NGF receptors are expressed on various cell types,^43,44^ to elucidate the direct effects of neutrophil-derived NGF on TRPV1^+^ nociceptive ingrowth during bone healing, we conditionally knocked down the *Ntrk1* gene, which encodes the NGF receptor neurotrophic tyrosine kinase receptor type 1 (TrkA), in TRPV1^+^ nociceptive neurons that innervate the femurs (Extended data Fig. 3g). The efficiency of the gene interference was confirmed via RT-PCR of DRGs (Extended data Fig. 3h) and immunofluorescence of healing femurs (Extended data Fig. 3i). We found that conditional knockdown of *Ntrk1* in TRPV1^+^ neurons mitigated neutrophil-mediated nociceptive innervation, and exogenous NGF administration failed to reverse the impairment of neurogenesis in targeted *Ntrk1* knockdown mice (Fig. 2i). Additionally, knockdown of the *Ntrk1* gene in DRGs isolated from TRPV1-Cre mice blocked the ability of day 5 NEU CM to promote neurite outgrowth and Ca2^+^ influx (Fig. 2j, k). Overall, these results proved that neutrophils initiated nociceptive axonogenesis via NGF-TrkA axis.

### Characterization of neurotrophin-synthesizing neutrophils

**Fig. 3:**
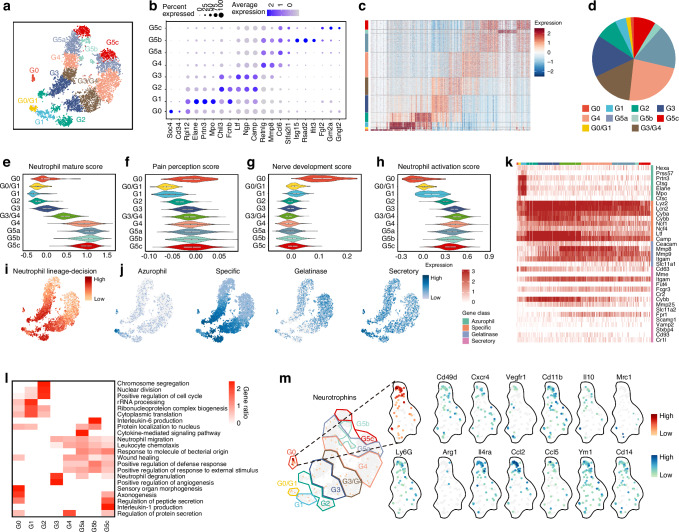
Characterization of neurotrophin-secreting neutrophils. **a** t-SNE of NEU + MP from the regenerating bone on day 5, colored by cell type. Expression of unique genes specifically distinguished each cluster and associated them with G0 (Sox4, CD34, Rpl12), G1 (Elane, Prtn3, Mpo), G2 (Chil3, Fcnb), G3 (Ltf, Ngp, Camp), G4 (Retnlg, Mmp8), G5a (Ccl6, Stfa2l1), G5b (Isg15, Rsad2, Ifit3), G5c (Fgl2, Gm2a, Gngt2) and two transition subcluster including G0/G1 and G3/G4 sharing the signature genes of two clusters, respectively. **b** Dot plot showing the scaled expression of selected signature genes for each cluster, colored by the average expression of each gene in each cluster scaled across all clusters. Dot size represents the percentage of cells in each cluster with more than one read of the corresponding gene. **c** Heatmap showing the row-scaled expression of the 20 highest DEGs (Bonferroni-corrected *P* values < 0.05; Student’s t-test) per cluster for all neutrophils. **d** Proportions of the neutrophil clusters. Violin plot of neutrophil maturation score (**e**), pain perception (**f**), nerve development (**g**) and neutrophil activation scores (**h**) for each cluster. t-SNE of neutrophils expressing neutrophil lineage-decision genes (Egr1, Fosb, Jun, Gata2, Gata1, Gfi1, Cebpa, Cebpe, Per3, Ets1, Irf8, Klf4, Zeb2 and Cybb) (**i**) and various granule genes including Azurophil (primary) granules, specific (secondary) granules, gelatinase (tertiary) granules and Secretory (quaternary) granules (**j**). **k** Heatmap showing the expression of neutrophil granule-related genes for all neutrophils. **l** Gene Ontology analysis of DEGs for each cluster. Selected Gene Ontology terms with Benjamini–Hochberg-corrected *P* values < 0.05 (one-sided Fisher’s exact test) are shown and colored by gene ratio. Colored areas indicate density distribution of data. **m** t-SNE of neutrophils expressing neurotrophin and alternatively activated genes

We next dissected the heterogenous neutrophil-related populations (myeloid progenitors and neutrophils) at single-cell resolution to identify the origin of neurotrophins. According to known gene signatures reported by Xie et al.^45^ 21 signature genes were adopted to cluster partitioned differentiating and mature neutrophils into 8 clusters and 2 transition clusters as G0-G5, with substantial differential gene expression (Fig. 3a–c). G3-G4 populations, which exhibited a relative mature characteristic based on the expression of genes related to neutrophil differentiation and maturation, made up more than half of the total population, whereas cells in earlier phase of neutrophil maturation, including G0-G1, represented only a small fraction (Fig. 3d, e). Gene Ontology score analysis showed that the nociceptive innervation-related genes mainly enriched in G0 neutrophils, which exhibited a low activation level (Fig. 3f-h). With the expression of important neutrophil lineage-decision genes^46^ (Fig. 3i), neurotrophin-producing neutrophils (Fig. 3m) predominantly expressed genes encoding primary (azurophil) granule, such as Mpo and Elane, as well as those encoding secondary (specific) granule, including Ltf and Camp, similar to previously described neutrophil precursors (NeuPs)^46–48^ (Fig. 3j, k). Moreover, like NeuPs, cell cycle-related genes were also enriched in this subcluster, indicating strong proliferative capacity (Fig. 3l). Consistently, ring-shaped and kidney-shaped nuclei were also observed in Ly6G^+^ NGF^+^ cells at injured sites, sharing the nuclei morphology characteristics with NeuPs, (Fig. 2e). Moreover, we compared the neurotrophin-expressed neutrophils identified in our study with previously reported pro-regenerative or anti-inflammation neutrophil subpopulations^49–53^ (Table 1, Fig. 3m). Although not completely matching any of the subsets listed below, this subpopulation showed substantial overlap with Il4ra⁺ and Ccl2 ^high^ cells, partially sharing the markers of a neuroregenerative neutrophil subset, as well as a tumor-associated anti-inflammatory cluster.

**Table. 1:** Properties and markers of previously reported neutrophils with alternative activation-like phenotype

Properties	marker	citation
Proangiogenic	CD49d^+^CXCR4^+^VEGFR1^+^	^49^
Anti-inflammatory	CD11b^+^CD49d^−^IL-10^+^	^50^
Pro-regenerative	Ly6G^low^, CD14^hi^, Arg1^+^, Mrc1^+^ and Il4r^+^	^51^
Anti-inflammation	Arg1^hi^, Ccl2^hi^, Ccl5^hi^	^52^
Anti-inflammation	Ym1^+^, CD206^+^	^53^

### Nociceptors promote osteoblastic LXA4 biosynthesis by autophagy to dampen neutrophilic inflammation

**Fig. 4:**
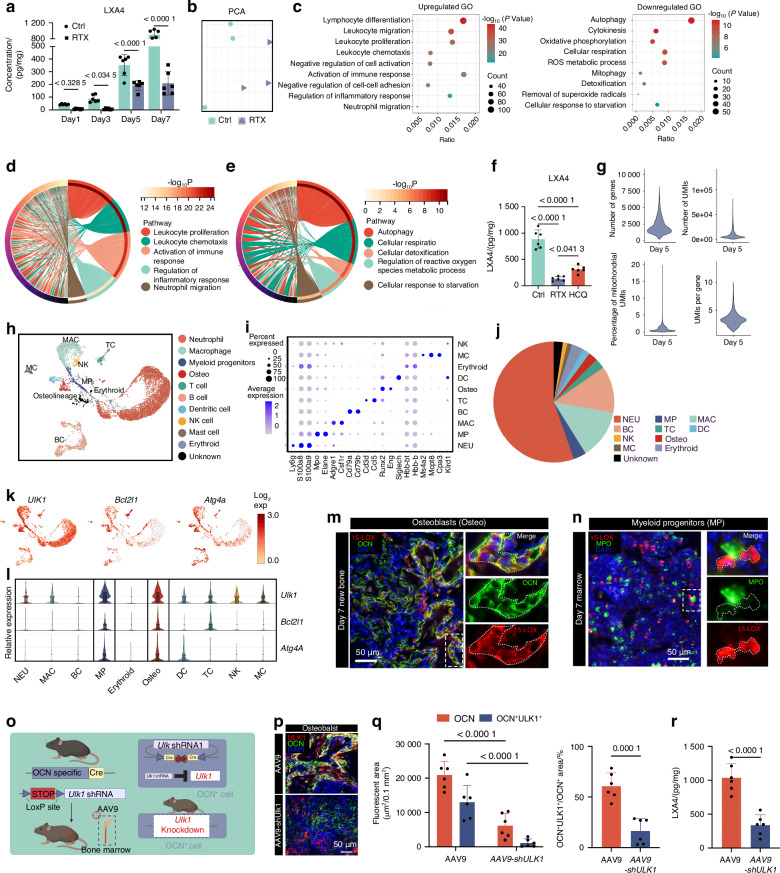
Nociceptors promote osteoblastic LXA4 biosynthesis by autophagy to dampen neutrophilic inflammation. **a** LXA4 levels in healing tissue following femoral injury (*n* = 6 femurs per time point). **b** PCA of transcriptional profiles. **c** GO enrichment analysis of the DEGs. **d**, **e** Circos diagram displaying intersection of different biological process in upregulated (**d**) and downregulated genes (**e**). **f** LXA4 levels in bone healing tissue isolated from Ctrl, HCQ-treated and RTX-treated mice on day 7 (*n* = 6 femurs per group). **g** Violin plots of the number of genes, number of UMIs, mitochondria count percentage, and UMI per gene of all QC-passed cells in day 7. **h** UMAP of 13,646 cells from the regenerating bone colored by cell type, Expression of unique genes specifically distinguished each cluster and associated them with neutrophil (Neu) (Ly6g, S100a8 and S100a9), myeloid progenitor (MP) (Mpo and Elane), macrophage (MAC) (Csf1r,Adgre1), B cell (BC)(Cd79a and Cd79b), T cells (T cell) (Cd3d and Ccl5), and dendritic cell (DC) (Siglech), osteolineage cell (Runx2 and Eng), mast cell (MC) (Ms4a2, Mcpt8 and Cpa3), NK cell (NK) (Klrd1) and erythroid (Hbb-bt and Hbb-bs), respectively. **i** Dot plot showing the scaled expression of selected signature genes for each cluster. **j** The proportion of each cell lineages from day 5. **k** UMAP plot showing the expression of three most significantly changed autophagy-related genes in bulk RNA-seq. **l** Relative expression of these three genes in all ten types of cells. **m**, **n** Representative images of the 15-LOX co-localization analysis with osteoblast (**m**) or myeloid progenitors (**n**). **o** Schematic diagram of conditional knockdown of Ulk1 gene in OCN^+^ osteoblasts. **p**, **q** The efficiency of conditioned knockdown was varied by representative images (**p**) and semi-quantative analysis of immunostaining of OCN (green) and ULK1 (red) in the femoral healing tissue on day 7 (*n* = 6 femurs per group) (**q**). Scale bars, 50 μm. **r** LXA4 levels in bone healing tissue isolated from AAV-9 and AAV9-shUlk1 treated OCN-cre mice on day 7 (*n* = 6 femurs per group). Data are mean ± s.e.m. and are representative of at least three independent experiments. *P* values determined by (**a**, **q** left) two-way ANOVA, **f** one-way ANOVA with Tukey tests and (**q** right, **r**) Student’s *t*-test

We next ask how nociceptive innervation stop acute neutrophilic inflammation during bone healing. The resolution of inflammation is now known as a temporally coordinated, active program directed by specialized pro-resolving mediators (SPMs).^54^ LXA4, the lead member of SPMs,^55,56^ is biosynthesized locally during inflammation and serves as a potent stop signal for neutrophil infiltration.^57,58^ We confirmed a significant increase of LXA4 level during healing process and it was suppressed by RTX treatment (Fig. 4a). However, arachidonate 15-lipoxygenase (15-LOX), which is necessary for LXA4 biosynthesis, was not abundant in nociceptors (Extended data Fig. 4a), suggesting that most of the LXA4 might not directly originate from nociceptors. For in-depth analysis, we performed bulk RNA sequencing on femurs of DMSO or RTX-treated mice on postinjury day 7. PCA revealed well-separated gene expression between two groups (Fig. 4b). No significant change in genes involved in LXA4 biosynthesis, including *Alox5*, *Alox12* and *Alox15*,^55^ was yet observed (Extended data Fig. 4b). Instead, GO enrichment analysis showed that nociceptive denervation inhibited autophagy and the response to oxidative stress, accompanied by markedly activated immune response (Fig. 4c). The Circos diagram further revealed an aberrant upregulation of neutrophilic inflammation (Fig. 4d). Besides, downregulated genes enriched in autophagy also partially overlapped with those involved in ROS metabolic process (Fig. 4e), demonstrating the intricate link between these two processes. Autophagy is a protective mechanism to maintain cellular functional under noxious stimuli, such as excessive oxidative stress caused by respiratory burst from neutrophils.^59–61^ We deduced that abnormalities in autophagy in the denervated mice might account for the impaired production of LXA4 in cells under excessive oxidative stress. Mice treated with hydroxychloroquine (HCQ), an FDA-approved autophagy inhibitor,^62,63^ exhibited decreased LXA4 levels, like denervated mice (Fig. 4f). This finding suggested that autophagy is necessary for nociceptor-mediated LXA4 secretion.

Next, we performed scRNA-seq on postinjury day 7 to identify the potential downstream targets of nociceptors. (Fig. 4g-l). After rigorous quality control, we obtained 13 646 high-quality cells with an average of 1 819 genes per cell profiled, resulting in a total of 26 016 mouse genes detected in all cells (Fig. 4g). After dimension reduction and graph-based clustering, we identified 10 major cell populations (Fig. 4h-j). UMAP of scRNA-seq indicated that three autophagy-related genes that significantly downregulated in bulk RNAseq of RTX treated mice, including *Ulk1*, *Atg4a* and *Bcl2l1* (Extended data Fig. 4b), mainly expressed in osteo-lineage cells (Osteo) and myeloid progenitors (MP) (Fig. 4k, l). Co-localization analysis of the markers of these two cell types with 15-LOX revealed a significant 15-LOX expression in OCN^+^ cells instead of MPO^+^ cells (Fig. 4m, n). Furthermore, we conditionally knocked down the *Ulk1* gene, which encodes Unc-51-like autophagy-activating kinase 1 (ULK1), a serine/threonine protein kinase that plays a crucial role in autophagy, in OCN^+^ osteoblasts (Fig. 4o). The osteoblast-specific ULK1 downregulation was confirmed via immunofluorescence (Fig. 4p, q). Unsurprisingly, the conditioned interference of autophagy in osteoblasts significantly inhibited the level of LXA4 in injured sites (Fig. 4r). These results collectively indicated that nociceptors activated autophagy in osteoblast to promote LXA4 biosynthesis, thereby facilitating inflammation resolution.

### Nociceptors rewire inflammatory microenvironment via CGRP

**Fig. 5:**
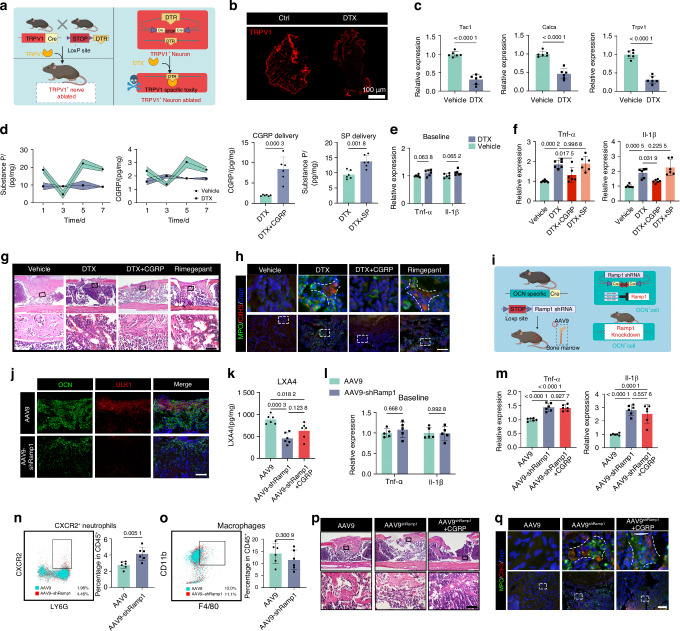
Nociceptors rewire inflammatory microenvironment via CGRP. **a** Schematic diagram of genetically ablating TRPV1^+^nociceptive nerves. **b**, **c** The efficiency of genetically ablation was verified according to representative images of immunostaining of TRPV1 (red) (**b**) and canonical genes expression in DRGs isolated from DMSO- or DTX-treated TRPV1-iDTR mice at steady state (*n* = 6 DRGs per group) (**c**). Scale bars, 100 μm. **d** (left)The local levels of neurotransmitter CGRP and SP in femurs isolated from indicated groups at different time points of bone healing (*n* = 6 femurs per time point). (right) Cgrp/Sp level at injured sites 24 h after topical administration of exogenous neuronal peptides on day5. Expression of Inflammation associated genes in femurs with sham operation (**e**) or bone defects (**f**) in indicated groups (*n* = 6 femurs per group). Representative images of H&E staining (**g**) and immunostaining (**h**) of NETs marker MPO (green) and CitH3 (red) of the femoral defects on day14. Scale bars, 100 μm. **i** Schematic representation of the conditional knockdown of the Ramp1 gene in osteoblasts. **j** Representative images of immunostaining of autophagy marker ULK1 (red) and osteoblasts (OCN) on the 7th day post femoral injury. Scale bars, 100 μm. **k** LXA4 levels in healing tissue isolated from OCN-cre mice on day 5 (*n* = 6 femurs per group). **l**, **m** Inflammation associated gene expression in femurs isolated from OCN-cre mice with sham operation (**l**) or femoral defects (**m**) on day 7 (*n* = 6 femurs per group). **n**, **o** Flow cytometry analysis of femoral defects isolated from AAV9- or AAV9shRamp1-treated OCN-cre mice on day7 post bone injury (*n* = 6 femurs per group). **p**−**q** H&E staining (**p**) and immunofluorescence (**q**) of NETs marker MPO (green) and CitH3 (red) of the femoral defects on day14. Scale bars, 100 μm. Data are mean ± s.e.m. and are representative of at least three independent experiments. P values determined by Student’s t-test (**c**, **o**) two-way ANOVA (**e**, **l**) and one-way ANOVA with Tukey tests (**f**, **k**, **m**)

The potential mediators through which nociceptors potentiate osteoblastic LXA4 biosynthesis was then investigated. TRPV1-iDTR mice were adopted for genetic ablation of TRPV1^+^ nociceptors^64^ (Fig. 5a) and ablation of TRPV1^+^ neurons in the DRG under diphtheriatoxin (DTX) treatment was confirmed (Fig. 5b, c). Calcitonin gene-related peptide (CGRP) and substance P (SP) are major neuropeptides by which nociceptors regulate host defense in response to noxious stimuli.^65^ DTX administration led to sharp decrease of CGRP and SP levels at injured sites (Fig. 5d), as well as imbalance between pro-inflammatory (CD45^+^Ly6G^+^CXCR2^+^)^38^ and anti-inflammatory (CD45^+^Ly6G^+^CD206^+^)^39,52,66^ neutrophils (Extended data Fig. 4c-e). Then we investigated whether the administration of SP or CGRP reversed the uncontrolled inflammation caused by TRPV1^+^ nociceptive denervation. The delivery efficiency was confirmed a day after topical administration of exogenous CGRP and SP (Fig. 5d). The unaltered expression of inflammatory genes suggested that DTX injection did not disrupt inflammatory homeostasis in the healthy state (Fig. 5e). TRPV1^+^ neuron ablation increased *Il-1β* and *Tnf-α* gene expression in the healing femurs, which can be inhibited by the delivery of CGRP rather than SP (Fig. 5f). Furthermore, delivery of exogenous CGRP reduced the infiltration of inflammatory cells and extensive NETs resulting from DTX injection, which was antagonized by Rimegepant, a CGRP receptor antagonist^67^ (Fig. 5g, h). These results suggested that neurogenic CGRP rewired the inflammatory environment during bone healing.

To confirm the role of CGRP in osteoblastic autophagy and LXA4 synthesis, we conducted an osteoblast-specific knockdown of the CGRP receptor, receptor activity-modifying protein 1 (RAMP1)^68^ (Fig. 5i). The efficiency of targeted *Ramp1* gene interference was verified both in vitro and in vivo (Extended data Fig. 4f, g). *Ramp1* knockdown substantially decreased the expression of autophagy marker ULK1 in osteoblasts (Fig. 5j), as well as decreased LXA4 level at injured sites, which was not reversed by CGRP (Fig. 5k). As the results, knockdown of *Ramp1*in osteoblasts did not result in any fluctuation in inflammatory cytokines in sham operation group (Fig. 5l), whereas led to an abnormal upregulation of *Il-1β* and *Tnf-α* expression in injured femurs, and these effects were not reversed by CGRP delivery (Fig. 5m). AAV9^sh*Ramp1*^ treatment significantly increased the proportion of pro-inflammatory neutrophils (CD45^+^Ly6G^+^CXCR2^+^) (Fig. 5n, o). Furthermore, AAV9^sh*Ramp1*^ infection led to extensive inflammatory cell infiltration (Fig. 5p) and a dispersed distribution of CitH3^+^MPO^+^ NETs (Fig. 5q) at injured sites, neither of which could be effectively controlled by the exogenous addition of CGRP (Fig. 5p, q). These results proved that osteoblasts acted as effectors downstream of neuronal CGRP for LXA4 production.

### CGRP-triggered autophagy rescues ROS-blocked LXA4 synthesis

**Fig. 6:**
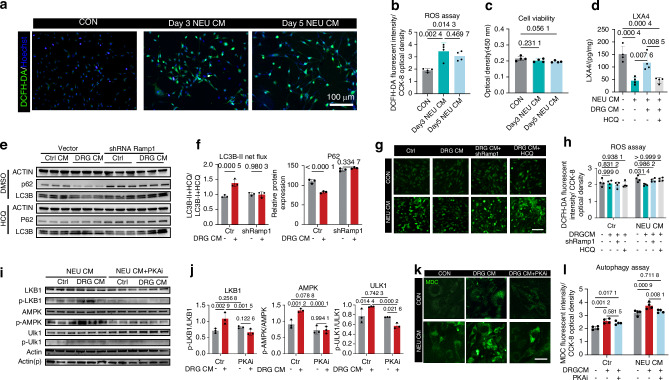
CGRP-triggered autophagy rescues ROS-blocked LXA4 synthesis. **a** Representative images (left) and **b** mean fluorescent intensity (right) of ROS in osteoblasts detected by DCFH-DA probe under indicated culture conditions for 4 h (*n* = 4 biologically independent samples per group). Scale bars, 100 μm. **c** Osteoblasts cell viability measured by CCK8 under indicated culture conditions for 12 h (*n* = 4 biologically independent samples per group). **d** LXA4 level in osteoblasts measured by ELISA under indicated culture conditions for 12 h (*n* = 4 biologically independent samples per group). **e**, **f** LC3B and p62 levels and the net flux of LC3B-II in osteoblasts under indicated culture conditions for 12 h (*n* = 3 biologically independent samples per group). **g**, **h** Representative images and mean fluorescent intensity of ROS in osteoblasts detected by DCFH-DA probe under indicated culture conditions for 4 h (*n* = 4 biologically independent samples per group). Scale bars, 50 μm. **i**, **j** WB analysis of the LKB1/AMPK/ULK1 pathway in osteoblasts under indicated culture conditions for 12 h (*n* = 3 biologically independent samples per group). **k**, **l** MDC staining for detecting autophagosomes in osteoblasts under indicated culture conditions for 12 h (*n* = 4 biologically independent samples per group). Scale bars, 10 μm. Data are mean ± s.e.m. and are representative of at least three independent experiments. *P* values determined by two-way ANOVA, except for (**b**, **c**) one-way ANOVA with Tukey tests

Considering the intricate link between autophagy and ROS metabolism revealed by RNA-sequencing (Fig. 4e), we hypothesized that neuronal CGRP-mediated autophagy rescued the osteoblastic LXA4 production impaired by ROS. Normally, ROS in injured areas mainly produced by neutrophil respiratory burst.^59^ Likewise, NEU CM elevated the intracellular ROS level in osteoblasts (Fig. 6a, b). Although NEU CM-induced oxidative stress was not sufficient to trigger cell death (Fig. 6c), it significantly decreased LXA4 secretion of osteoblasts. This effect was reversed by conditioned medium from activated DRG (DRG CM) (Fig. 6d), which was antagonized by HCQ (Fig. 6d). Treating osteoblasts with DRG CM significantly increased LC3BII levels and reduced P62 levels under NEU CM-induced oxidative stress. Notably, the increase in autophagic flux was significantly blocked by *Ramp1* interference (Fig. 6e, f). Moreover, either HCQ treatment or *Ramp1* knockdown inhibited DRG CM-mediated ROS clearance (Fig. 6g, h). These findings supported our hypothesis that neurogenic CGRP facilitates osteoblastic LXA4 biosynthesis under excessive ROS from neutrophils.

We further investigated the signal transduction underlying the CGRP-induced autophagy. AMP-activated protein kinase (AMPK) induces autophagy by phosphorylating and activating ULK1, which requires the regulation of liver kinase B1 (LKB1).^69,70^ Accordingly, addition of DRG CM increased LKB1 activity, consequently resulting in phosphorylation of AMPK and activation of ULK1 in osteoblasts (Fig. 6i, j). The CGRP-RAMP1 interaction leads to increased levels of the second messenger cAMP and activation of the cAMP dependent protein kinase PKA.^71^ We therefore incubated osteoblasts with cAMP analogue Rp-8-CPT-cAMP, a selective PKA inhibitor (PKAi), before they were treated with DRG CM. The results showed that the PKAi almost completely suppressed the activation of the LKB1/AMPK/ULK1 cascade by DRG CM (Fig. 6i, j), and antagonized the formation of autophagosomes (Fig. 6k, l). Taken together, these findings suggested that the PKA/LKB1/AMPK/ULK1 cascade underlies the CGRP-induced activation of autophagy in osteoblasts.

### Autophagy limits 5-LOX nuclear location for LXA4 synthesis

**Fig. 7:**
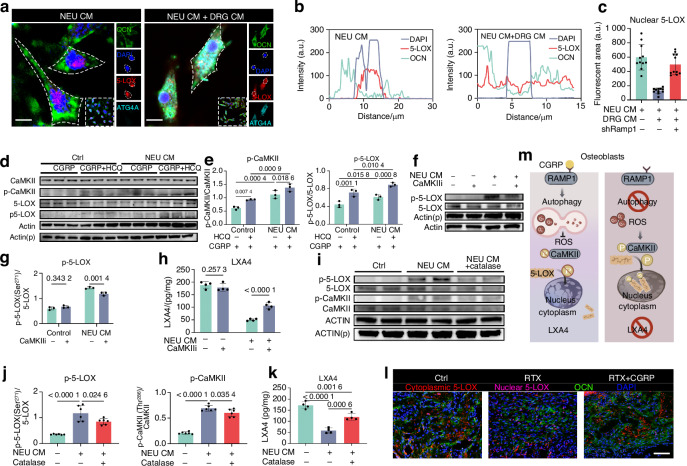
Autophagy limits 5-LOX nuclear location for LXA4 synthesis. **a** Representative images of immunostaining and **b** colocalization and **c** semi-quantative analysis of ATG4A (cyan), 5-LOX (red) and DAPI (blue) in osteoblast (OCN^+^, green) under indicated culture conditions for 12 h (*n* = 12 random areas from 4 biologically independent samples per group). Scale bars, 10 μm. **d**, **e** WB analysis of the phosphorylation levels of CaMKII and 5-LOX in osteoblasts under indicated culture conditions for 12 h (*n* = 3 biologically independent samples per group). **f**, **g** WB analysis of the Ser271 phosphorylation levels of 5-LOX in osteoblasts treated by DMSO or CaMKIIi (HY-18271) with/without NEU CM. **h** LXA4 level in osteoblasts under indicated culture conditions for 12 h (*n* = 4 biologically independent samples per group). **i**, **j** WB analysis of the Thr286 phosphorylation level of CaMKII and Ser271 phosphorylation level of 5-LOX in osteoblasts under indicated culture conditions for 12 h. **k** LXA4 level in osteoblasts under indicated culture conditions for 12 h (*n* = 4 biologically independent samples per group). **l** Representative images of cytoplastic 5-LOX (red) and nuclear 5-LOX (Magenta) in OCN^+^ osteoblasts (Green) in femurs isolated from mice treated with DMSO or RTX, Scale bars, 100 μm. **m** Schematic diagram of CGRP mediated LXA4 biosynthesis. Data are mean ± s.e.m. and are representative of at least three independent experiments. *P* values determined by (**c**) Student’s t-test, (**e**, **g**, **h**) two-way ANOVA and (**j**, **k**) one-way ANOVA with Tukey tests

We next tried to find the missing pieces in autophagy-mediated LXA4 biosynthesis. 5-lipoxygenase (5-LOX), often in combination with 12/15-lipoxygenases, is involved in the biosynthesis of LXA4. However, expression of enzyme-encoding genes was not affected by denervation (Extended data Fig. 4b). Notably, previous studies found that the subcellular location of 5-LOX affects balance of arachidonic acid metabolism^72,73^: CaMKII-mediated Ser^271^ phosphorylation of 5-LOX facilitated its nuclear retention, which increases pro-inflammatory leukotriene production, while the translocation of 5-LOX from the nucleus to the cytoplasm favors lipoxin biosynthesis. Likewise, the addition of DRG CM in osteoblasts incubated with NEU CM effectively inhibited the nuclear retention of 5-LOX, which was abrogated by *Ramp1* knockdown (Fig. 7a-c). CGRP reduced the phosphorylation of CaMKII at Thr^286^ and 5-LOX at Ser^271^, which was antagonized by autophagy inhibition (Fig. 7d, e). Consistently, the phosphorylation of 5-LOX was inhibited by CaMKIIi, which also promoted LXA4 biosynthesis (Fig. 7f−h). Of note, CaMKII is reported to be activated by ROS,^74^ while catalase treatment^75^ inhibited the phosphorylation of CaMKII and 5-LOX by NEU CM, leading to increased osteoblastic LXA4 production (Fig. 7i−k). Importantly, the delivery of CGRP to the RTX-treated mice significantly reduced the amount of 5-LOX^+^ area located in nucleus (Fig. 7l). Altogether, these findings suggested that CGRP-mediated autophagy eliminated intracellular ROS to inhibit the CaMKII-triggered 5-LOX phosphorylation, thereby blocking the nuclear retention of 5-LOX and potentiating the LXA4 production. (Fig. 7m).

### Evoking nociceptors boosts alveolar healing in diabetic mice

**Fig. 8:**
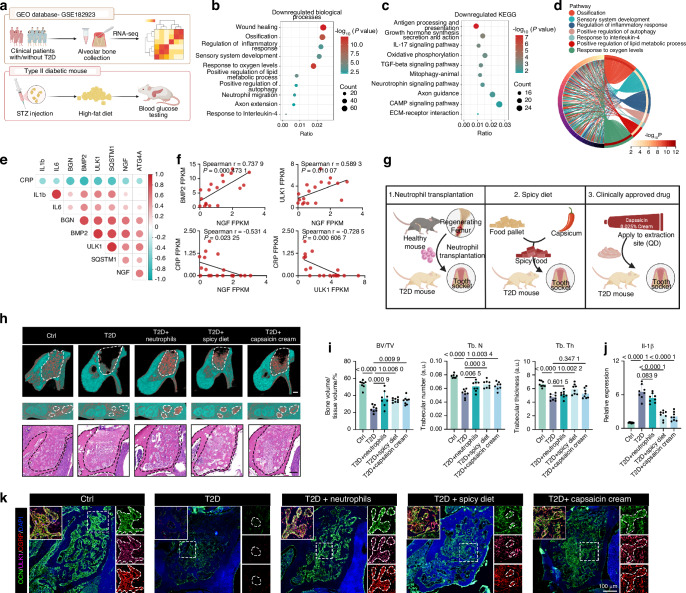
Evoking nociceptors promotes alveolar bone healing in diabetic mice. **a** Schematic representation of transcriptome data analysis of the GEO database and the construction of a mouse model of type 2 diabetes. **b**−**c** GO and KEGG enrichment analysis of the DEGs. **d** Circos diagram displaying gene intersections in different biological processes. **e**, **f** Spearman correlation analysis of the expression of genes related to nerve regeneration, osteogenesis, autophagy, and inflammation. **g** Schematic representation of three therapeutic strategies for healing tooth extraction sockets in type 2 diabetes mouse model. **h**−**i** Micro-CT reconstructions, H&E staining, and quantitative analysis of BV/TV, Tb.N and trabecular thickness (Tb.Th) of mouse tooth extraction sockets (TESs) on day 9 post-tooth extraction (*n* = 8 TESs per group). Scale bars represent 100 μm. **j** Il-1β expression in TESs isolated from control or type II diabetic mice with/without treatment (*n* = 8 TESs per group). **k** Representative images of OCN^+^ osteoblasts, the autophagy marker ATG4A and the nociceptive transmitter CGRP in healthy and type II diabetic mice with/without treatment. Scale bars, 100 μm. Data are mean ± s.e.m. and are representative of at least three independent experiments. *P* values determined by one-way ANOVA with Tukey tests (**i**, **j**) and Spearman’s rank-order correlation coefficient (**f**)

Considering critical role of nociceptor-mediated negative feedback regulation on inflammation, we furthered explored whether activation of nociceptors favored the healing of inflammatory bone defects. Clinically, alveolar sockets after tooth extraction in patients with diabetes are common refractory bone defects due to dysregulation of inflammation.^76–78^ To explore the potential relevance between nociceptors and inflammation during alveolar healing of diabetic patients, we analyzed the gene transcriptional profiles in alveolar bone collected from healthy individuals and diabetic patients (GSE182923, Ayilavarapu et al.) (Fig. 8a). Consistent with our findings, human genomic data revealed that genes downregulated in the context of diabetes were significantly associated with autophagy, sensory system development, inflammation modulation, ROS metabolism, and bone formation (Fig. 8b-d). Considering that most genes in DRG was not included in alveolar bones, *Ngf* expression was adopted to indirectly assess the level of sensory innervation. Spearman correlation analysis showed that *Ngf* expression was significantly positively related to the osteogenic marker gene *Bmp-2* and the autophagic gene *Ulk1*. Conversely, a strong negative correlation was found between the expression of *Ngf* and the inflammation marker C-reactive protein(*Crp*).^79^ Additionally, the expression of *Ulk1* was also significantly negatively correlated with *Crp* expression (Fig. 8e, f). These findings revealed that samples with diminished nociceptive innervation displayed reduced autophagy levels and increased inflammation, suggesting that the regeneration or activation of nociceptive nerves could be a strategy target for managing abnormal inflammation in diabetic alveolar bone.

Next, we constructed a tooth extraction socket model in diabetic mice for verification^80^ (Fig. 8a). Given the downregulation of *Ngf* and suppressed nociceptive function in diabetic patients, we devised three therapeutic strategies to promote extraction socket healing in type II diabetic mice by regenerating or controllably activating nociceptive nerves (Fig. 8g). Briefly, we either used an adoptive transfer model in which neutrophils from the injured bone tissues of healthy mice were delivered into the extraction sockets of diabetic mice, exploiting neutrophil-derived NGF, or tried to activate the release of neuronal CGRP via the ingestion of food containing capsaicin, or local administration of Capsaicin cream, an FDA-approved TRPV1 agonist for neuropathic pain treatment. Micro-CT analysis indicated that all three strategies increased the alveolar bone regeneration inhibited by diabetes (Fig. 8h, i). As expected, all three strategies notably decreased the level of the inflammatory cytokine Il-1β (Fig. 8j) and increased the extent of CGRP secretion, promoted autophagy within the diabetic alveolar sockets, and thereby potentiated bone healing (Fig. 8k). Overall, these results support the strategy of accelerating the inflammation-regeneration transition through the rehabilitation of neuroimmune interplay in diabetic alveolar bone defects.

## Discussion

Recent advances in neuroimmune interactions have revealed a critical role for sensory nerves in immune defense against noxious stimuli within highly innervated organs. However, the outcome of such interactions has been proven to be tissue specific. For example, JAK1 gain-of-function (GoF) variants in skin-innervating sensory nerves drive allergic inflammation, whereas JAK1 GoF from lung-innervating sensory nerves suppressed airway inflammation.^81^ Baral et al. reported that activation of lung-innervating nociceptors was lethal to patients with bacterial lung infections due to suppression of host immune response*s*,^82^ while pain-sensing nerves in skins were reported to promote wound healing via the regulation of neutrophils and macrophages.^13,83^ Bone tissue, with a high density of sensory innervation, exhibits a more complex immune composition, characterized by a greater diversity and abundance of immune cells within the bone marrow. Whether bone-innervating sensory nerves regulate inflammation after bone injury remains unclear. In this study, we have shown that postinjury nociceptive innervation elicited by neutrophils acted as negative feedback to safeguard inflammation-regeneration transition during bone healing. Specifically, we identified a previously unknown cluster of Il4ra^+^ and Ccl2^high^ neutrophils that promotes TRPV1^+^ nociceptors innervation to injured areas via NGF-TrkA axis. The neuronal CGRP, in turn, activated autophagy in osteoblasts, thereby pro-resolving mediator, LXA4 biosynthesis, rewiring the inflammatory microenvironment. By analyzing transcriptome profile of alveolar bones from diabetic patients, we found positive correlation among sensory dysregulation, impaired autophagy, and uncontrolled inflammation. Therapeutic strategies aimed at regenerating or activating nociceptors successfully rescued impaired alveolar healing in diabetic mice.

Macrophages have been previously reported to promote nerve sprouting through NGF during intra-periosteum bone formation and cranial bone defect healing.^9,37^ However, in the femoral bone defect model, we found that the neuroimmune interactions mainly took place between neutrophils and nociceptors. Depletion of neutrophils, other than macrophages, impaired the nociceptive ingrowth. Accordingly, ablation of TRPV1^+^ nociceptors exacerbated neutrophil infiltration, with a minor impact on macrophages, DCs and lymphocytes. Using scRNA-sequencing to dissect the heterogeneity of bone marrow neutrophils, a unique Il4ra^+^ Ccl2^high^ subpopulation of early-stage neutrophils, characterized by active proliferation similar to neutrophil progenitors,^46–48^ gave rise to the neurotrophins. We deduced that the heterogeneity of neutrophils contributed to the discrepancy in NGF derivation between our research and previous reports—unlike femurs, flat bones such as cranial bones contain only a limited amount of bone marrow components.^84^ Neutrophils infiltrated to periosteum or cranial bones are mainly mature neutrophils from peripheral blood, which are predominantly differentiated, mature cells with specialized anti-infection functions.^85,86^ Quite opposite, bone marrows of femurs are known as generating heterogenous neutrophils and consist of few macrophages.^87^ Nevertheless, the potential existence of other factors, such as alternative activation of neutrophils, warrants further research. Besides, although we employed multiple approaches to validate the interaction between nociceptors and neutrophils, lineage tracing using nociceptor- and neutrophil-specific reporter mice could provide more specific and accurate evidence of this post-injury neuro-immune interaction. Furthermore, lineage tracing techniques would facilitate the isolation of neurotrophin-synthesizing neutrophil subpopulation identified in this work for further characterization.

The effects of CGRP on the osteogenic differentiation have been widely investigated, yet CGRP-mediated immunoregulation of osteoblasts has rarely been reported. Here, we demonstrated that neurogenic CGRP activates autophagy in osteoblasts, hence alleviating the ROS-mediated phosphorylation of CaMKII, thereby leading to cytoplasmic localization of 5-LOX. Treatment of refractory wound in diabetic patients owing to dysregulated inflammation is a major challenge. In this study, we discovered that activating nociceptive nerves via a spicy diet or topically applying capsaicin cream mitigated inflammation and promoted alveolar healing in type II diabetic mice. Intriguingly, a study involving 200 million individuals in China indicated that a preference for spicy foods is associated with a lower risk of developing diabetes.^88^ The correlation between the immunoregulation of nociceptors and diabetes needs further investigation. Based on above results, our work might open a new avenue for inflammatory bone diseases treatment. Pain is a prevalent and frequently debilitating symptom experienced by individuals suffering from chronic inflammatory bone diseases, including osteoarthritis and rheumatoid arthritis. While the origin of this pain is multifactorial, current management strategies—such as the use of NSAIDs, opioids, and glucocorticoids—primarily target central and peripheral sensitization mechanisms.^89^ Despite careful clinical selection, the safety and efficacy of these drugs remain questionable due to their potential for undesirable side effects,^90^ prompting the need for alternative therapeutic options. Of note, increased nociceptor density represents another critical factor contributing to pain perception in chronic inflammatory bone diseases.^91^ In this study, we demonstrated that neurotrophin released by neutrophils promotes nociceptive nerve sprouting, highlighting neutrophil-derived neurotrophic signaling as a potential therapeutic target for pain management in inflammatory bone diseases. In addition, previous studies have shown that osteoblast-derived leukotrienes (LTs) produced via 5-LOX played a critical role in mediating pathological bone remodeling in arthritis.^92,93^ By contrast, activation of osteoblastic autophagy could shift the products of 5-LOX from LTs to LXA4. This “turning waste into value” strategy offers a novel strategy for protecting subchondral bone.

In the present study, we identified the immunoregulatory role of nociceptors in both femoral defect regeneration of healthy mice and extraction socket healing of diabetic mice, implying that such neuroimmune interaction might serve as an intrinsic negative feedback control of inflammation during bone healing. However, recent studies revealed that the immunomodulation by nociceptive nerves is quite complex and influenced by multiple factors, such as microbiota^15,94^ and the local microenvironment.^81^ Given diverse causes of bone inflammation (trauma, infection, and autoimmune diseases) and the dynamic osteoimmune microenvironment,^95^ the specific regulatory mechanisms of the neuroimmune crosstalk in bone healing under different conditions still require further investigation. In addition, we mainly focused on elucidating the role of neuro-immune interaction in the inflammation-regeneration transition during the early phase of bone defect repair. It is noteworthy that bone healing requires tight coordination of osteoblasts and osteoclasts, especially in the subsequent bone remodeling phase. Recent studies have revealed that osteoblast- or macrophage-derived PGE2 could activate EP4 in sensory nerves to regulate osteoclasts by suppressing sympathetic nervous activity via central nervous system.^8,96^ Moreover, increased Cx3cr1^+^ inflammatory osteoclasts within the bone callus delayed the bone healing via suppressing CGRP^+^TrkA^+^ sensory nerve–bone signaling exchange.^97^ These results indicated potential crosstalk between nociceptors and osteoclasts. Nevertheless, whether nociceptors could directly regulate the bioactivity of osteoclasts need further elucidation.

In summary, this study uncovered unrecognized negative feedback of inflammation, in which TRPV1^+^ nociceptive ingrowth driven by neutrophil derived NGF orchestrated the inflammation resolution by facilitating osteoblastic lipoxin A4 production during bone healing. We believe that identifying the immunomodulatory role of bone-innervating nociceptors can increase the clinical understanding of pain management strategies and accelerate the development of neuromodulation-based therapies for chronic inflammatory bone diseases.

## Materials and methods

A full description of the “Materials and methods” is provided in SI Appendix, including in vivo drug administration, local Delivery of CGRP/SP, behavioral experiments, Micro-CT analysis, histology and IHC/ICC staining, bulk RNA sequencing, Single cell RNA sequencing, ELISA, flowcytometry, isolation and culture of cells, ROS assay, MDC assay, RNA isolation, qPCR and WB analysis.

### Statistics

All data were tested for normality by the Kolmogorov-Smirnoff test. Unpaired two-tailed Student’s *t*-test (parametric data) was used for simple comparisons. For comparisons of more than two groups, data were analyzed by one-way analysis of variance (ANOVA) test, followed by Tukey’s post hoc tests or two-way ANOVA test. Numerical data are graphed in bar charts with mean ± s.e.m. Nonlinear regression (curve fit) is utilized to predict the trends in changes of immunofluorescence area at various time points, and Spearman’s rank-order correlation coefficient is employed to detect the correlation between gene expressions during the bone healing process. The significance level was set at *P* < 0.05, with all P values reported in the figures (GraphPad Prism).

### Animals

TRPV1-Cre (C001295, Cyagen, USA), OCN-Cre (C001025, Cyagen), and ROSA26-LSL-DTR^fl/stop/fl^ (C001189, Cyagen) mice were bred at specific pathogen-free facilities. C57BL/6 mice were purchased from Shanghai Jiesijie Laboratory Animals Company (China). For selective depletion of TRPV1-expressing nociceptors, TRPV1-Cre mice were crossbred with ROSA26-LSL-DTR^fl/stop/fl^ mice. All mice were aged between 10 and 16 weeks, age-matched for each experimental setup, and maintained on a 12 h light-dark cycle with ad libitum access to food and water. Only male mice were included in the experimental. Littermates were randomly allocated to different experimental conditions.

The type II diabetic mouse model was generated by feeding 8-week-old male C57BL/6 mice a high-fat diet (high-fat diet with 60 kal%, FB-12492, WuxiFanbo Biotechnology Co., Ltd., China) for 4 weeks, followed by a fasting injection of low-dose streptozotocin (STZ, 30 mg/kg, S0130, Sigma‒Aldrich, Missouri, USA). The success of the type II diabetic model was verified by fasting glucose levels consistently over 11.1 mmol/L for 2 weeks. Afterward, all the mice were fed a regular diet.

The care and use of the animals were in strict compliance with the guidelines approved by the Animal Welfare Committee of the Shanghai Jiao Tong University School of Medicine, Affiliated Ninth People’s Hospital (approval number SH9H-2024-A1009-1), adhering to recognized standards for animal welfare. Research methodologies were designed to minimize animal distress and suffering.

### Neutrophil and macrophage depletion

Intraperitoneal injection of rat anti-mouse Ly6G antibody (clone 1A8; Bio X Cell, USA) was performed to deplete femoral neutrophils. An initial dose of 200 μg per mouse was administered two days before surgery, followed by 100 μg every other day until euthanasia. The control mice received an equivalent dose of rat IgG. The effectiveness of neutrophil (CD45^+^/Ly6G^+^) depletion in femurs was confirmed through flow cytometry.

Intraperitoneal injection of clodronate liposomes (40337ES08, Yeasen, China) was performed to deplete femoral macrophages. A dose of 200 μL per mouse was administered every day from day 3 before surgery to euthanasia. The effectiveness of macrophage depletion in repairing femurs was confirmed through immunostaining.

### TRPV1^+^ nociceptors ablation

According to previously reported protocols,^12,98,99^ RTX (AN125100U, CFW Laboratories, USA) was prepared in a saline solution with 1% ethanol and administrated for in vivo pharmacological ablation of TRPV1^+^ nociceptors. Mice were anesthetized with isoflurane and the dorsal skin was carefully shaved and disinfected. A precise 1 cm incision was made to expose the vertebral column. 10 µL PBS containing 50 ng RTX was administered at L3–L5 DRG using a microsyringe. For complete delivery of the compounds, the microsyringe was subsequently flushed with 10 µL of saline immediately after each injection. This procedure was consistently repeated over three consecutive days and succeeded by a seven-day recovery period before surgical interventions.

For systemic genetic TRPV1^+^ nociceptor ablation, TRPV1-iDTR transgenic mice were intraperitoneally injected with 100 ng DTX (D0564, Sigma‒Aldrich, USA) daily for seven consecutive days.^64^ The mice selected for this procedure were aged between 10 and 12 weeks, ensuring uniformity in the experimental conditions.

### Construction of lentiviral and adeno-associated viral vectors (AAV) for gene interference

For generation of lentiviral vectors for gene silencing, three shRNAs targeting the *Ramp1* gene were synthesized, cloned, and inserted into the lentiviral vector PGMLV-SC5-GFP (Genomeditech, China). This vector was propagated in osteoblasts for 7 days, and the interference efficacy was evaluated via qPCR. The sequences of the *Ramp1* shRNAs used were as follows: Ramp1-shRNA1: CCCTGACTATGGGACTCTCAT; Ramp1-shRNA2:GGAGAACATGGAGACTATTGG; Ramp1-shRNA3: GGACAGATTCTTCATCGCTGT

For targeted gene silencing in vivo, selected shRNA were inserted into the AAV9 vector GPAAV-HU6-MCS-CMV-eGFP-WPRE (PGMAAV-10261, Genomeditech, China) utilizing the double-inversion orientation (DIO) system. This setup allows Cre recombinase-dependent shRNA expression in specific cell types, ensuring precise gene silencing. *Ramp1* shRNA and *Ulk1* shRNA were cloned and inserted into the AAV9 vector for injection into bone marrow cavity and a *Ntrk1* shRNA was cloned and inserted into the same AAV9 vector for DRG infection. The sequences used were as follows: Ramp1-shRNA1: CCCTGACTATGGGACTCTCAT; Ntrk1-shRNA: TCTATAGCACAGACTATTACC; Ulk1-shRNA: CGCTTCTTTCTGGACAAACAA

### Injection of AAV9 to bone marrow cavity or spinal canal

For targeted gene silence in bone marrow, viral vectors were prepared at a concentration of 1 × 10^12^ viral genomes in a 5 μL volume and injected into the bone marrow through a bone marrow aspirate needle as previously validated.^100^ Mice received 5 μL of the specified vector every 3 weeks for a total of 12 weeks. The efficacy of gene interference was assessed by IHC staining.

For targeted gene silence in TRPV1^+^ neurons within DRG, viral vectors (either AAV9 or AAV9 *Ntrk1*) were injected as previously validated.^101^ After anesthetized, the dorsal skin of mice was carefully shaved and disinfected. For exposure of the vertebral column, a 1 cm incision was made at the junction of the femur and the spine. Viral vectors (either AAV9 or AAV9-sh*Ntrk1* were prepared at a concentration of 5 × 10^11^ viral genomes in a total volume of 10 μL. This solution was administered through a micro syringe into the spinal cavity across segments L3–L5. The efficacy of gene interference was assessed by IHC staining 3 weeks after injection.

### Construction of murine femoral diaphysis defect model

Cortical defects were performed on middle femoral diaphysis in adult mice as adapted and modified from previously validated protocols.^23–26^ Mice were anesthetized with isoflurane gas for the duration of the experiment. The skin around the hindlimb was carefully shaved and then disinfected with alcohol. A 1 cm longitudinal incision was made along the lateral aspect of the femur. The muscles anterior and posterior to the femur were gently separated with ophthalmic scissors to expose the mid-diaphysis of the femur. A dental implant system equipped with a 1.5 mm diameter bur was used to drill perpendicularly into the mid-diaphysis at a speed of 2 000 r/min until the opposite bone wall was reached. Saline was applied continuously to cool the drilling site, and extensive saline irrigation was applied post-drilling to remove bone debris. Subsequently, the muscle and skin layers were sutured closed, and the mouse was then placed in a recovery chamber until fully awake. Femurs were harvested 0−21 days after surgery.

### Construction of murine molar extraction socket healing model

Mice were anesthetized using isoflurane gas. The first molars on both sides of the maxilla were extracted with dental forceps. In the neutrophil treatment group, previously isolated neutrophils were injected directly into the extraction sockets at a concentration of 2.5 × 10^7^ cells/mL according, for a total volume of 2 μL. The concentration was adapted from a previously validated neutrophil adoptive transfer protocol.^102^ The sites were then allowed to clot before the mice were placed in a recovery chamber until they regained consciousness. In the spicy diet group, a specialized diet containing 0.01% (mass ratio) capsaicin was prepared by incorporating it into standard mouse feed,^103^ and the mice were maintained on this diet until euthanasia. In the capsaicin cream treatment group, 0.025% capsaicin cream (50488-1025, Alexso, USA) was applied to the extraction sites once daily after surgery. All mice were euthanized by cervical dislocation on the day 9 post-injury.

## Data and materials availability

All data are available in the main text or the supplementary materials. Additional data related to this paper may be requested from the authors.

## Supplementary information

Supplementary Information
