# Supplementary material for: Neutrophil-initiated nociceptive ingrowth orchestrates inflammation resolution to potentiate bone regeneration
Source: Bone Res. 2026 Jan 19;14:9. doi: 10.1038/s41413-025-00481-6 (PMC12816638; doi:10.1038/s41413-025-00481-6)
Supplement: Supplementary file 1 — Supplementary Information [file 41413_2025_481_MOESM1_ESM.docx]

**Supplementary Information**

Materials and methods

**In vivo drug administration**

For degradation of extracellular DNA and NETs, mice were treated with DNase I (1121MG010, Biofroxx, Germany) at 10 mg/kg once a day until 7 days post-injury ^1^. For inhibition of autophagy, 60 mg/kg HCQ (H0915, Sigma‒Aldrich, USA) was administered via daily intraperitoneal injection after surgery until euthanasia ^2,3^.

**Local Delivery of CGRP/SP**

To avoid possible off-target effects resulted from high drug concentration and systemic administration, we delivered drugs via locally percutaneous injection at injured sites ^4^. Results of ELISA indicated that both CGRP and SP began to increase at injured sites at 5 days post-injury (Fig. 5d), while the peak level of which are between 0.1-1ng/defect. Taking the diffusion and the metabolism of the drugs into consideration, we locally injected 1μg of either CGRP or SP once a day from 5 days post-injury to sacrification. Specifically, experimental groups consisted of 1μg CGRP (P10092, Novoprotein, China) or 1μg Substance P (P20366, Novoprotein, China) in 20 μl PBS, compared to control injection of 20 μl PBS, were administrated percutaneously on the surface of femoral defects. The dosage of the drugs delivery was supported by previous works ^5,6^.

**Behavioral experiments**

Gait analysis of C57BL/6 mice following a femoral defect or sham operation was conducted as adapted from previously validated protocols ^7-9^ using the VisuGait system (Shanghai Xin Luan MDT Infotech, China). The mice were acclimated to standard laboratory conditions prior to the initiation of the study to ensure behavioral consistency and minimize stress. Mice were allowed to traverse an enclosed walkway freely, ensuring an unimpeded analysis of their locomotor patterns. Key gait parameters were assessed accurately in the walkway equipped with high-precision sensor array. The analysis was initiated after recovery from surgical anesthesia, with evaluations conducted from day 0-13 to meticulously document pain progression during bone healing. The inverse ratio of ground contact time between the injured and noninjured limbs was quantitatively assessed as a marker of pain. Comprehensive gait metrics, including paw pressure, print area, stance and swing phases, duty cycle, stride length, and swing speed, were captured and analyzed.

Open field test was conducted as adapted from previously validated protocol ^10^ in a quiet environment. After femoral defect surgery, mice from both α-Ly6G group (intraperitoneal injection of rat anti-mouse Ly6G antibody before surgery) and control group (received an equivalent dose of rat IgG before surgery) were centrally placed within a 40 cm × 40 cm × 40 cm white, square arena. Mice were allowed with an unimpeded exploration over a period of 10 min on day 5. Behavioral observations were recorded using a high-definition video camera (Shanghai Xin Luan MDT Infotech, China). Quantitative analysis of locomotor activity including total distance, average velocity, duration of supportive standing, and time spent in designated zones of the enclosure, was conducted using specialized animal behavior analysis software (Shanghai Xin Luan MDT Infotech, China). Both the interior surfaces and the base of the arena were sterilized with 75% ethanol after each testing session.

**Micro-CT analysis**

For analysis of bone regeneration, specimens were scanned using a high-resolution micro-CT system (Skyscan1171, Bruker, USA). The femurs were centrally placed in the sample holder to guarantee even scanning. The scanning parameters included a 0.5 mm aluminum filter, a 50 kVp X-ray tube voltage, and an 18 μm pixel size. The collected projections were then reconstructed using the system's specialized software suite—NRecon, Dataviewer, CTvox, and CTan (Bruker, USA)—enabling thorough analysis. A grayscale threshold of 45 to 255 was set to outline bone tissue in the scans. This approach facilitated the creation of three-dimensional (3D) reconstructions for accurate assessment of bone architectural integrity. Quantitative analysis was conducted to evaluate crucial bone quality indicators, such as the BV/TV, Tb. Th, and Tb.N, providing in-depth insights into regenerative outcomes. For methodological consistency and data reliability, all scanning and analysis procedures were performed by the same technician. Additionally, the micro-CT system was calibrated before each use with a standardized phantom to ensure the precision and repeatability of the imaging results.

**Histology and IHC/ICC staining**

Fixed femurs were decalcified in 20% EDTA for up to one month. Then samples were processed for paraffin embedment (10- or 50-μm-thick sections). Longitudinal sections were mounted on adhesive slides. H&E staining was performed using standard protocols. For IHC/ICC analysis, samples were washed 3 times in PBS for 5 minutes. Then the sections or cells were first permeabilized with 0.5% Triton-X for 30 minutes. The cells, or the sections following antigen retrieval in citrate buffer (pH 6.0) with heat treatment, were blocked using a solution of 5% donkey serum and incubated with primary antibodies overnight at 4°C. The following day, samples were washed in PBS, incubated in the appropriate fluorescent secondary antibody and then mounted with antifade mounting medium with DAPI. Digital images of these sections were captured with ×10 to ×40 objectives using confocal microscopy (Sp8, Leica, Germany).

For staining with primary antibodies from the same species, the Multicolor Fluorescent Immunohistochemical Staining Kit (Absin, abs50012) was used. Briefly, the samples were subjected to microwave-assisted antigen retrieval in 1× solution, followed by endogenous peroxidase quenching with 3% H_2_O_2_. After being washed with TBST, the samples were blocked and incubated with primary antibodies and then with HRP-conjugated secondary antibodies. Amplification of fluorescent signals was achieved using TSA fluorescent dyes, and after microwave treatment to remove bound antibodies, steps were repeated for different primary antibodies. The samples were washed and stained with DAPI. Samples were mounted with antifade medium and imaged using fluorescence microscopy (Sp8, Leica, Germany). The semi-quantative analysis was processed using ImageJ software and the NeuronJ plugin.

The following antibodies were used for immunstaining: rabbit anti-TRPV1 (Novus, NB100-1617, 1:100 dilution), mouse anti-PGP9.5 (Abcam, ab8189, 1:50 dilution), rabbit anti-NF200 (Abclonal, A23495, 1:200 dilution), rabbit anti-CitH3 (Novus, NB100-57135, 1:2000 dilution), goat anti-MPO (Novus, AF3667, 15 μg/ml), rat anti-Ly6G (Novus, NBP2-00441, 1:200 dilution), mouse anti-CD68 (Abcam, ab955, 1:3000 dilution), mouse anti-OCN (Abcam, ab13418, 10 μg/ml), mouse anti-5-LOX (Abmart, MG256887,1:2000 dilution), rabbit anti-15-LOX(Abcam, abs244205,1:4000 dilution), rabbit anti-RAMP1(Absin, abs117175,1:200 dilution), rabbit anti-ULK1 (T56902, Abmart, 1:1,000 dilution), rabbit anti-CGRP(Abcam, ab47027, 1:200 dilution), and rabbit anti-NGF(Abcam, ab52918,1:100 dilution).

**Bulk RNA sequencing**

To instigate transcriptomic alterations after nociceptive denervation, C57BL/6 mice in Vehicle- or RTX- treated groups were subjected to femoral defect surgery and euthanized on day 7 post-injury. The harvested tissue included the femoral defect area along with approximately 1 mm of adjacent bone segments on both sides, encompassing bone tissue, bone marrow, and reparative tissue. Samples were preserved at -20°C. The extraction of RNA was performed using TRIzol® (Invitrogen, USA), and the integrity of the RNA was assessed via agarose gel electrophoresis, a NanoPhotometer (Implen, Germany), and an Agilent 2100 bioanalyzer (Agilent Technologies, USA). Following mRNA enrichment and fragmentation, library construction was performed using NEB Buffer (New England Biolabs, USA) and the TruSeq™ Kit (Illumina, USA). These libraries were quantified and sequenced on an Illumina HiSeq2000 platform using the TruSeq SBS Kit v3-HS. The sequencing data were subjected to quality control with FastQC, adapter sequences were trimmed using Trimmomatic, HISAT2 was used for reads alignment, and HTSeq was used for quantifying gene expression. DESeq2 was used for differential expression analysis, and DAVID was used for enrichment analysis. Data visualization was facilitated through R and Bioconductor packages.

**Single cell RNA sequencing.**

Femoral healing tissue isolated from 4 femurs was collected at specific time point. Single-cell suspensions from 4 repeated samples were mixed, filtered through 40 μm cell strainers, lysed with red blood cells and suspended in PBS containing 0.05% BSA. Cell viability and counting were evaluated with trypan blue by microscopy, and samples with viabilities >85% were used for sequencing. Libraries were constructed using the Single Cell 3′ Library Kit V3 (10× Genomics, USA). Transcriptome profiles of individual cells were determined by 10× Genomics-based droplet sequencing. About 25000 cells were loaded to capture 13000 cells. Then, indexed complementary DNA (cDNA) libraries were sequenced with paired end reads on an Illumina NovaSeq 6000 (Illumina). Cell Ranger (https://10xgenomics.com/) was used to process Chromium single-cell 3′ RNA-seq output ^11^.

Once the gene-by-cell data matrix was generated, poor quality cells were excluded. Only genes expressed in three or more cells were used for further analysis. Cells were also discarded if their mitochondrial gene percentages were over 20%. The data were natural log transformed and normalized for scaling the sequencing depth to a total of 1 × 10^4^ molecules per cell, followed by regressing out the number of UMIs using Seurat package (version 5.0.0). Seurat downstream-analysis steps included dimensionality reduction (principal-component analysis) and UMAP, standard unsupervised clustering and the discovery of differentially expressed cell-type-specific markers. Differential gene expression analyses to identify cell-type-specific genes were performed using Loupe Browser software. Clusters that do not match major cell markers within the bone are labeled as Unknown.

Functional scores were defined as the average normalized expression of corresponding genes ^12^. Briefly, Z scores were calculated by scaling the normalized expression of a gene across all cells. Gene weights were set to either 1 or −1 to reflect positive or negative relationships. The gene listed in neutrophil maturation signature was from ref. ^12^. Other functional signatures were derived from the Gene Ontology database: Pain perception (GO:0019233), Nerve development (GO:0021675) and neutrophil activation (GO:0042119).

**ELISA**

Samples were initially immersed in PBS supplemented with protease inhibitor (Yeasen, China) and subjected to mechanical disruption with ophthalmic scissors, followed by homogenization using a tissue lyser (F6/10, Jingxin, China) and ultrasonication (VCX130, Sonics, USA) to ensure thorough cell lysis and protein solubilization. After homogenization, the samples were centrifuged at 12,000 rpm for 15 min in a refrigerated centrifuge to separate the supernatant for analysis. ELISAs were employed for the quantification of the neurotrophins NGF, BDNF, GDNF, NT-3 and NT-4 (EK1286, EK5128, EK14220, EK15835, EK15838, Signalway Antibody, USA), the nociceptive neurotransmitters SP and CGRP (ab3181 and ab3227, Abmart, China) and the pro-resolving mediator LXA4 (EK10003, Signalway Antibody, USA). These assays were conducted according to the manufacturers' protocols. Absorbance readings were obtained using a multifunctional microplate reader (Spark, Tecan, Switzerland), and concentrations were deduced from standard curves, ensuring the methodology's conciseness and adherence to professional standards.

**Flow cytometry.**

Following euthanasia, the femoral defect segments were promptly immersed in ice-cold PBS. The tissues were finely minced using ophthalmic scissors and incubated in an enzymatic cocktail (deoxyribonuclease I at 300 U/ml, collagenase type IV at 500 U/ml, and hyaluronidase at 2700 U/ml; all from Biofroxx, Germany) at 37°C with shaking at 90 rpm for 1 h. After incubation, the tissues were further dissociated through a cell strainer, and the filtrate was centrifuged at 500×g for 5 min to collect the cells. The cells were then resuspended in PBS to obtain single-cell suspensions for immunofluorescence staining. The following reagents and antibodies were used for immunofluorescence staining: Fixable Viability Stain 700 (564997, BD,USA), purified rat anti-CD16/CD32 mouse Fc Blocker (553141, BD), BV510 rat anti-CD45 (563891, BD), PE hamster anti-CD29 (562801, BD), PerCP-Cy5.5 rat anti-CD44 (560570, BD), BV 421 rat anti-Ly-6G (127627, BioLegend, USA), PE-Cy7 rat anti-mouse CD182 (CXCR2) (149315, BioLegend), FITC rat anti-CD11b (101205, BioLegend), APC rat anti-F4/80 (123115, BioLegend), PE-Cy7 rat anti-CD86 (105013, BioLegend), AF647 rat anti-CD206 (141711, BioLegend), BV711 hamster anti-CD11c (117349, BioLegend), PE-Cy7 hamster anti-mouse TCR β chain (109221, BioLegend), and BV605 rat anti-CD45R/B220 (103243, BioLegend). Specific populations, including neutrophils (CD45^+^Ly6G^+^), mature neutrophils (CD45^+^Ly6G^+^CD11b^+^), activated neutrophils (CD45^+^Ly6G^+^CXCR2^+^) and anti-inflammatory (CD45^+^Ly6G^+^CD206^+^) neutrophils ^13-15^, macrophages (CD45^+^F4/80^+^CD11b^+^), B cells (CD45^+^B220^+^), T cells (CD45^+^TCRβ^+^), DCs (CD45^+^CD11c^+^) ^16^, and mesenchymal stem cells (CD45^-^CD29^+^CD44^+^), were identified and quantified. Analysis was conducted using a FACS Fortessa cytometer (BD Biosciences) and FlowJo software.

**Isolation and culture of neutrophils**

The MojoSort Mouse Ly6-G Selection Kit (480123, BioLegend, USA) was used for neutrophil isolation from the femoral healing tissue of 12-week-old mice 3 or 5 days postoperatively. Humane euthanasia was conducted via cervical dislocation. Subsequently, bone healing tissues were collected by flushing femurs with heparinized culture medium using a 1 ml syringe. The tissues were finely minced using ophthalmic scissors and incubated in an enzymatic cocktail (deoxyribonuclease I at 300 U/ml, collagenase type IV at 500 U/ml, and hyaluronidase at 2700 U/ml; all from Biofroxx, Germany) at 37°C with shaking at 90 rpm for 1 h. After incubation, the tissues were further dissociated through a cell strainer, and the filtrate was centrifuged at 500 × g for 5 min to collect the cells. The cell mixture was then subjected to biotin-antibody cocktail and streptavidin nanobead treatment, according to the kit's instructions, to specifically tag Ly6-G-positive neutrophils. A magnetic separation technique was employed to segregate the bead-bound neutrophils from the cell mixture. After the isolation process, the neutrophils were maintained in complete α-MEM, after which they were prepared for further experimental analysis.

NEU CM was used to investigate the influence of neutrophils in bone healing tissue on axonal extension, Ca^2+^ influx in nociceptive neurons and oxidative stress induction in osteoblasts. After isolation from femurs on day 3 or 5 post-bone injury, all neutrophils were washed with PBS three times and cultured with 2.5 ml of DMEM for 24 h. The CM was then collected, centrifuged at 1500×g for 5 min, filtered through a 0.22-mm filter, and diluted 1:1.5 with fresh medium to ensure adequate nutrients for DRG neurons or osteoblasts. For boiled CM, the CM was boiled at 100°C for 7 min, cooled to room temperature, filtered through a 0.22-mm filter, and diluted 1:1.5. Nucleic acid degradation was achieved by treating the CM with boiling treatment, 2 mg/ml RNase (1341MG025, Biofroxx, Germany) or 1 unit/ml DNase I (1121MG010, Biofroxx, Germany) and incubating it at 37°C for 1 h before dilution.

**Isolation and culture of** **DRG neurons**

As previously validated protocol ^17^, DRGs were isolated from 3-4 week-old mice following humane euthanasia, after which the L3-L5 spinal segments were carefully harvested. The DRGs were then precisely removed from the dorsal spine area and placed immediately into ice-cold MEM. After washing in fresh medium, the ganglia were subjected to 30 min of digestion at 37°C in 0.05% trypsin-EDTA. The digested cells were seeded on poly-l-lysine-coated 6-well plates filled with neurobasal media, and DRGs from individual mice were assigned to each well. The following day, the medium was refreshed and enriched with 10 μM cytosine arabinoside (ARA-C) to inhibit non-neuronal cell proliferation, with subsequent medium changes occurring every other day.

For verification of the role of the NGF-TrkA axis in axon extension, DRGs from the L3-L5 segments of *Trpv1*-Cre mice were transduced with either AAV9 or AAV9 shRNA targeting *Ntrk1* (*shNtrk1*). DRG neurons were then isolated and plated in 24-well culture plates and maintained under previously specified conditions for three days before being fixed with 4% paraformaldehyde.

For calcium imaging, DRG neurons were incubated with 5 mM Fluo3-AM (40703ES, Yesean, China) in neurobasal media at 37°C for 30 min, followed by two washes and imaging in 2 ml of Krebs buffer. Upon application of specific stimuli, images were captured using an Sp8 fluorescence microscope (Leica, Germany) with a 488 nm excitation wavelength, and the fluorescence intensity was quantified using a Spark multifunctional microplate reader (Tecan, Switzerland). The specific stimuli used were performed as adapted and modified by previous validation ^18^: NEU CM, 1 μM capsaicin (Mecklin, China), and 40 mM KCl (Mecklin, China).

For determination of the influence of nociceptors on osteoblasts, DRG CM was utilized under various culture conditions. Briefly, DRG neurons were preincubated with NEU CM for 20 h. Then, the cells were incubated in 2.5 ml of DMEM for 72 h. After the incubation period, the DRG CM was harvested, centrifuged at 1500×g for 5 min, and filtered through a 0.22-μm filter. The CM was then diluted with fresh medium at a ratio of 1:1.5 to provide sufficient nutrients for osteoblast culture.

**Osteoblast induction and culture**

As previously validated ^19^, mouse femurs were isolated, washed and digested in 0.1% collagenase type I (1904MG100, Biofroxx, Germany) and 0.2% dispase II (40104ES60, Yeasen, China) in PBS for 10 min, and this process was repeated six times. The fractions were collected in culture medium consisting of complete α-MEM. For in vitro osteoblast differentiation, primary calvarial cells were induced with 100 mM ascorbic acid, 5 mM β-glycerophosphate and 10 nM dexamethasone for 14 days. The culture medium was changed every third day.

For analysis of the impact of nociceptor derived CGRP, osteoblasts in the DRG CM+sh*Ramp1* group were pre-infected with a lentivirus encoding shRNA targeting *Ramp1*. Additionally, for modulation of intracellular signaling pathways, the following specific treatment conditions were adopted: NEU CM, catalase (HY-P7744, MCE, USA) at 10 ng/ml, CGRP (Novoprotein, China) at 10 μM, DRG CM, sh*Ramp1*, HCQ (HY-B1370, MCE, USA) at 100 μM, CaMKIIi (HY-18271, MCE, USA) at 0.06 μM, and PKAi (Rp-8-CPT-cAMP, Cayman Chemical, USA) pretreatment for 1 h at 10 μM.

**ROS assay**

2',7'-Dichlorodihydrofluorescein diacetate (DCFH-DA) (S0033S, Beyotime, China) was used to quantify intracellular ROS levels in osteoblasts. After cultured under specific conditions for 12 h, osteoblasts were incubated with 10 μM DCFH-DA for 30 min at 37°C in an atmosphere containing 5% CO_2_. After incubation, the cells were washed three times with PBS to eliminate any unbound DCFH-DA. Representative fluorescence images were captured using a Leica Sp8 fluorescence microscope (Leica, Germany) at excitation/emission wavelengths of 488/525 nm. The fluorescence intensity was measured using a Spark multifunctional microplate reader (Tecan, Switzerland). The specific treatment conditions were as follows: NEU CM, HCQ (HY-B1370, MCE, USA) at 100 μM, DRG CM, and sh*Ramp1* (osteoblasts preinfected with a lentivirus encoding shRNA targeting the *Ramp1* gene).

.

**MDC assay**

To observe autophagosomes, we employed MDC staining (C3019, Yeasen, China). After the cells were subjected to the specified experimental conditions for 12 h, they were stained with MDC (50 μM) for 15 min to visualize autophagosomes. Staining steps were carried out at 37°C. After staining, the cells were washed with PBS and immediately analyzed. Fluorescence observation was performed using a fluorescence microscope (SP8, Leica, Germany), with MDC excitation/emission at 335/508 nm. Additionally, the fluorescence intensity of MDC was quantified using a Spark multifunctional microplate reader (Tecan, Switzerland). The specific treatment conditions were as follows: NEU CM, DRG CM, and sh*Ramp1* (osteoblasts pre-infected with a lentivirus encoding a shRNA targeting *the Ramp1* gene).

**RNA isolation and qPCR**

Following euthanasia, cells or femoral healing tissue isolated from mice were immediately homogenized in RNAiso Plus reagent (T9108, TaKaRa, Japan) for total RNA extraction. The extracted RNA was then reverse transcribed into cDNA using the PrimeScript™ RT Reagent Kit (RR036A, TaKaRa, Japan). Quantitative reverse transcription PCR (qRT‒PCR) analyses were conducted on a LightCycler® 480 II system (Roche, Switzerland) utilizing iQ™ SYBR® Green Supermix (Bio-Rad). β-Actin served as the internal control for normalization. The RT‒PCR primers used were as follows: mouse *Cxcr2,* 5′- ATGCCCTCTATTCTGCCAGAT-3′ and 5′- GTGCTCCGGTTGTATATAAGATGAC-3′; mouse *Gap43,* 5′- TGGTGTCAAGCCGGAAGATAA-3′ and 5′- GCTGGTGCATCACCCTTCT-3′; mouse *Il1β,* 5′- GCAACTGTTCCTGAACTCAACT-3′ and 5′- ATCTTTTGGGGTCCGTCAACT-3′; mouse *Il10,* 5′- GCTCTTACTGACTGGCATGAG-3′ and 5′- CGCAGCTCTAGGAGCATGTG-3′; mouse *Alp,* 5′- CCAACTCTTTTGTGCCAGAGA-3′ and 5′- GGCTACATTGGTGTTGAGCTTTT-3′; mouse *Calca,* 5′- GAGGGCTCTAGCTTGGACAG-3′ and 5′- AAGGTGTGAAACTTGTTGAGGT-3′; mouse *Tac1*, 5′- AAGCGGGATGCTGATTCCTC-3′ and 5′- TCTTTCGTAGTTCTGCATTGCG-3′; mouse *Trpv1* 5′- CATCTTCACCACGGCTGCTTAC-3′ and 5′- CAGACAGGATCTCTCCAGTGAC-3′; mouse *Tnfα* 5′- CCCTCACACTCAGATCATCTTCT-3′ and 5′- GCTACGACGTGGGCTACAG-3′; mouse *Ifnγ* 5′- ATGAACGCTACACACTGCATC-3′ and 5′- CCATCCTTTTGCCAGTTCCTC-3′; mouse *Ngf* 5′- CCAGTGAAATTAGGCTCCCTG-3′ and 5′- CCTTGGCAAAACCTTTATTGGG-3′; mouse *Bdnf* 5′- GGCTGACACTTTTGAGCACGTC-3′ and 5′- CTCCAAAGGCACTTGACTGCTG-3′; mouse *Ntf3* 5′- GGAGTTTGCCGGAAGACTCTC-3′ and 5′- GGGTGCTCTGGTAATTTTCCTTA-3′.

**WB analysis**

For protein analysis, the samples were initially lysed in RIPA buffer supplemented with protease and phosphatase inhibitors (20124ES, 20109ES05, Yeasen, China) and maintained on ice. For complete cell lysis and protein solubilization, the samples were then subjected to ultrasonication (VCX130, Sonics, USA). Following ultrasonication, the samples were centrifuged at 12,000 rpm for 15 min at 4°C in a refrigerated centrifuge. The protein concentrations of the supernatants were determined using a bicinchoninic acid (BCA) protein assay. Identical amounts of protein from each sample were electrophoresed on 4–20% Tris‒glycine gradient gels (36256ES10, Yeasen, China) and then transferred to PVDF membranes. These membranes were then subjected to incubation with the following primary antibodies: mouse anti-β-actin (30101ES, Yeasen, 1:10,000 dilution), mouse anti-β-actin (30101ES, Yeasen, 1:10,000 dilution), rabbit anti-CaMKII (12666-2-AP, Proteintech, 1:1,000 dilution), rabbit anti-T286 phospho-CaMKII alpha (T59748S, Abmart, 1:1,000 dilution), rabbit anti-S428 phospho-LKB1 (PA2122s, Yeasen, 1:1,000 dilution), rabbit anti-LKB1 (10746-1-AP, Proteintech, 1:1,000 dilution), rabbit anti-S271 phospho-5-LOX (PS06242, Abmart, 1:1,000 dilution), rabbit anti-5-LOX (MG247013, Abmart, 1:2,000 dilution), rabbit anti-AMPK alpha 1 (T55326F, Abmart, 1:1,000 dilution), rabbit anti-T172 phospho-AMPK (TA3423F, Abmart, 1:1,000 dilution), rabbit anti-p62/SQSTM1 (T55546F, Abmart, 1:1,000 dilution), rabbit anti-ULK1 (T56902, Abmart, 1:1,000 dilution), rabbit anti-S757 phospho-ULK1 (AP0736, ABclonal, 1:1,000 dilution), and rabbit anti-LC3b (T55992F, Abmart, 1:1,000 dilution). The antibodies were incubated at 4 °C overnight, followed by incubation with appropriate HRP-conjugated secondary antibodies, goat anti-rabbit IgG (H+L)-HRP conjugate (170-6515, Bio-Rad, 1:10,000 dilution), and goat anti-mouse IgG (H+L)-HRP conjugate (170-6516, Bio-Rad, 1:10,000 dilution), for 1 h at room temperature. The blots were developed with enhanced chemiluminescence substrate (36224ES, Yeasen, China). Blots were quantified using ImageJ software.

Methods References

1 Demkow, U. Molecular Mechanisms of Neutrophil Extracellular Trap (NETs) Degradation. Int. J. Mol. Sci. 24 (2023). https://doi.org/10.3390/ijms24054896

2 Mauthe, M. et al. Chloroquine inhibits autophagic flux by decreasing autophagosome-lysosome fusion. Autophagy 14, 1435-1455 (2018). https://doi.org/10.1080/15548627.2018.1474314

3 McAfee, Q. et al. Autophagy inhibitor Lys05 has single-agent antitumor activity and reproduces the phenotype of a genetic autophagy deficiency. Proc. Natl. Acad. Sci. U. S. A. 109, 8253-8258 (2012). https://doi.org/10.1073/pnas.1118193109

4 Bielecki, T., Gazdzik, T. S. & Szczepanski, T. Benefit of percutaneous injection of autologous platelet-leukocyte-rich gel in patients with delayed union and nonunion. Eur. Surg. Res. 40, 289-296 (2008). https://doi.org/10.1159/000114967

5 Siddiqui, Y. D. et al. Substance P aggravates ligature-induced periodontitis in mice. Front. Immunol. 14, 1099017 (2023). https://doi.org/10.3389/fimmu.2023.1099017

6 Lu, Y. Z. et al. CGRP sensory neurons promote tissue healing via neutrophils and macrophages. Nature 628, 604-641 (2024). https://doi.org/10.1038/s41586-024-07237-y

7 Kwon, S. B. et al. A machine learning-based diagnostic model associated with knee osteoarthritis severity. Sci. Rep. 10, 15743 (2020). https://doi.org/10.1038/s41598-020-72941-4

8 Blazquez-Carmona, P. et al. Gait analysis: An effective tool to mechanically monitor the bone regeneration of critical-sized defects in tissue engineering applications. PLoS One 18, e0296510 (2023). https://doi.org/10.1371/journal.pone.0296510

9 Coulthard a, Barbara J. Pleuvry b, Mike Brewster c, Kevin L. Wilson d, & a, T. V. M. Gait analysis as an objective measure in a chronic pain model. *J Neurosci Methods.* 2002;116(2):197-213. doi:10.1016/s0165-0270(02)00042-0.

10 Jia, X. et al. High-intensity swimming alleviates nociception and neuroinflammation in a mouse model of chronic post-ischemia pain by activating the resolvin E1-chemerin receptor 23 axis in the spinal cord. Neural Regen Res 18, 2535-2544 (2023) https://doi.org/10.4103/1673-5374.371373

11 Park, J. et al. Single-cell transcriptomics of the mouse kidney reveals potential cellular targets of kidney disease. Science (New York, N.Y.) 360, 758-763 (2018). https://doi.org/10.1126/science.aar2131

12 Xie, X. et al. Single-cell transcriptome profiling reveals neutrophil heterogeneity in homeostasis and infection. Nat. Immunol. 21, 1119-1133 (2020). https://doi.org/10.1038/s41590-020-0736-z

13 Min Young Park1, Hyung Sik Kim1, Ha Young Lee, Brian A. Zabel & Bae, Y.-S. Novel CD11b Gr-1 Sca-1 myeloid cells drive mortality in bacterial infection. Science Advance 6, eaax8820 (2020).

14 Jaillon, S. et al. Neutrophil diversity and plasticity in tumour progression and therapy. Nat Rev Cancer.  20, 485-503 (2020). https://doi.org/10.1038/s41568-020-0281-y

15 Fridlender, Z. G. et al. Polarization of tumor-associated neutrophil phenotype by TGF-beta: "N1" versus "N2" TAN. Cancer Cell 16, 183-194 (2009). https://doi.org/10.1016/j.ccr.2009.06.017

16 Silva, L. M. et al. Fibrin is a critical regulator of neutrophil effector function at the oral mucosal barrier. Science 374, eabl5450 (2021). https://doi.org/10.1126/science.abl5450

17 Richner, M., Jager, S. B., Siupka, P. & Vaegter, C. B. Hydraulic Extrusion of the Spinal Cord and Isolation of Dorsal Root Ganglia in Rodents. J Vis Exp (2017). https://doi.org/10.3791/55226

18 Yang, D. et al. Nociceptor neurons direct goblet cells via a CGRP-RAMP1 axis to drive mucus production and gut barrier protection. Cell 185, 4190-4205 e4125 (2022). https://doi.org/10.1016/j.cell.2022.09.024

19 Ishikawa, M. et al. Bone marrow plasma cells require P2RX4 to sense extracellular ATP. Nature 626, 1102-1107 (2024). https://doi.org/10.1038/s41586-024-07047-2

Extended data:

**
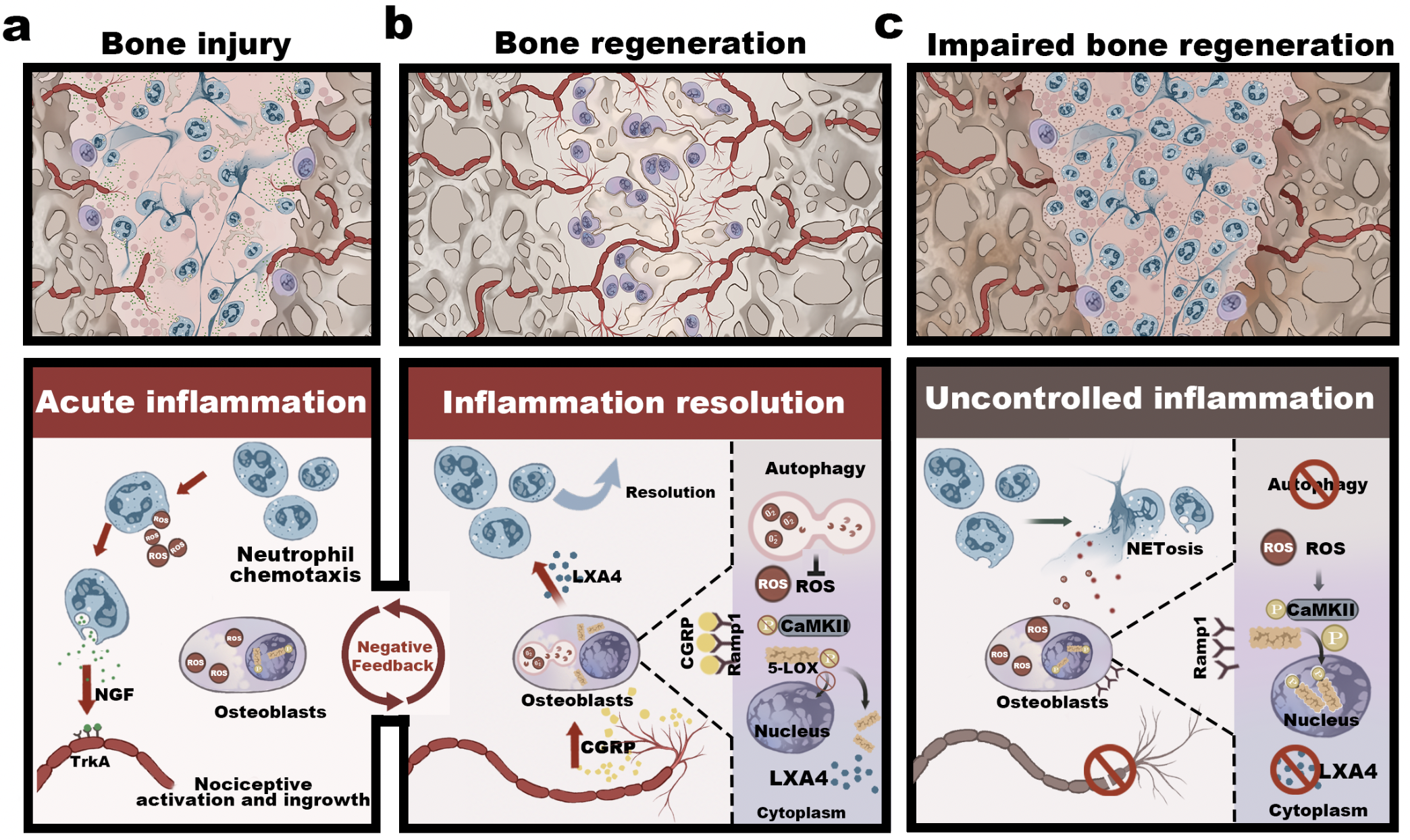
**

**Graphical abstract**

**
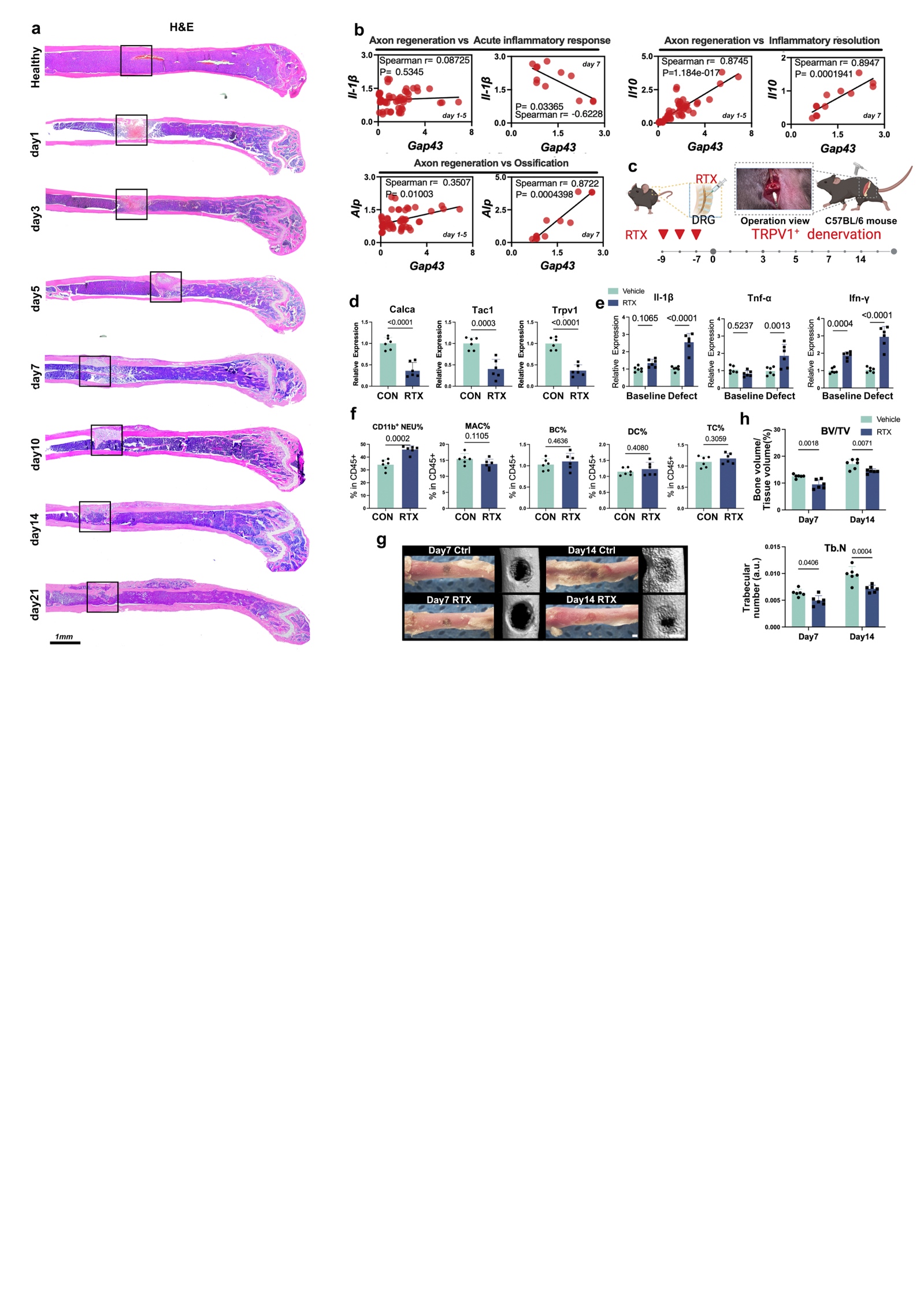
**

**Extended data Fig. 1 Nociceptive denervation leads to bone inflammation**

(a) Representative images of H&E-staining of regenerating bone at serial time points between days 1 and 21 after injury. The healing of this model consists of an inflammation stage characterized by inflammatory infiltration on day 1-3, a transition stage characterized by cell recruitment on day 5, a regeneration stage characterized by bone formation on day 7-14 and a remodeling stage characterized by morphological remodel of new bone on day 14-21. (b) Spearman correlation analysis among gene expression of axon regeneration marker Gap43, acute inflammation marker Il-1β, inflammatory resolution marker Il-10 and osteogenesis marker Alp on day1-5(n=53) and day7(n=12) during bone healing. (c) Schematic diagram of pharmacological ablation of TRPV1+ nociceptors in murine femoral diaphysis defect model. (d) The efficiency of pharmacological ablation of nociceptors was verified according to gene expression in DRGs isolated from the Vehicle- or RTX-treated C57BL/6 mice at the steady state (n=6 mice per group). (e) Inflammation-associated gene expression of the femoral defects in Vehicle- or RTX-treated C57BL/6 mice (n = 6 femurs per group). Scale bars, 1 mm. (f) Flow cytometry analysis of innate and adaptive immune cells in femoral defects isolated from Vehicle- or RTX-treated C57BL/6 Mice on day7 post bone injury (n= 6 femurs per group). (g) Representative images, micro-CT reconstructions and (h) quantative analysis of the femoral defects in Vehicle-treated or RTX-treated C57BL/6 mice (n = 6 femurs per group). Scale bars, 1 mm. Data are mean ± s.e.m. and are representative of at least four independent experiments. P values determined by (d, f) Student’s t-tests, (e, h) two-way ANOVA and (b) Spearman's rank-order correlation coefficient.

**
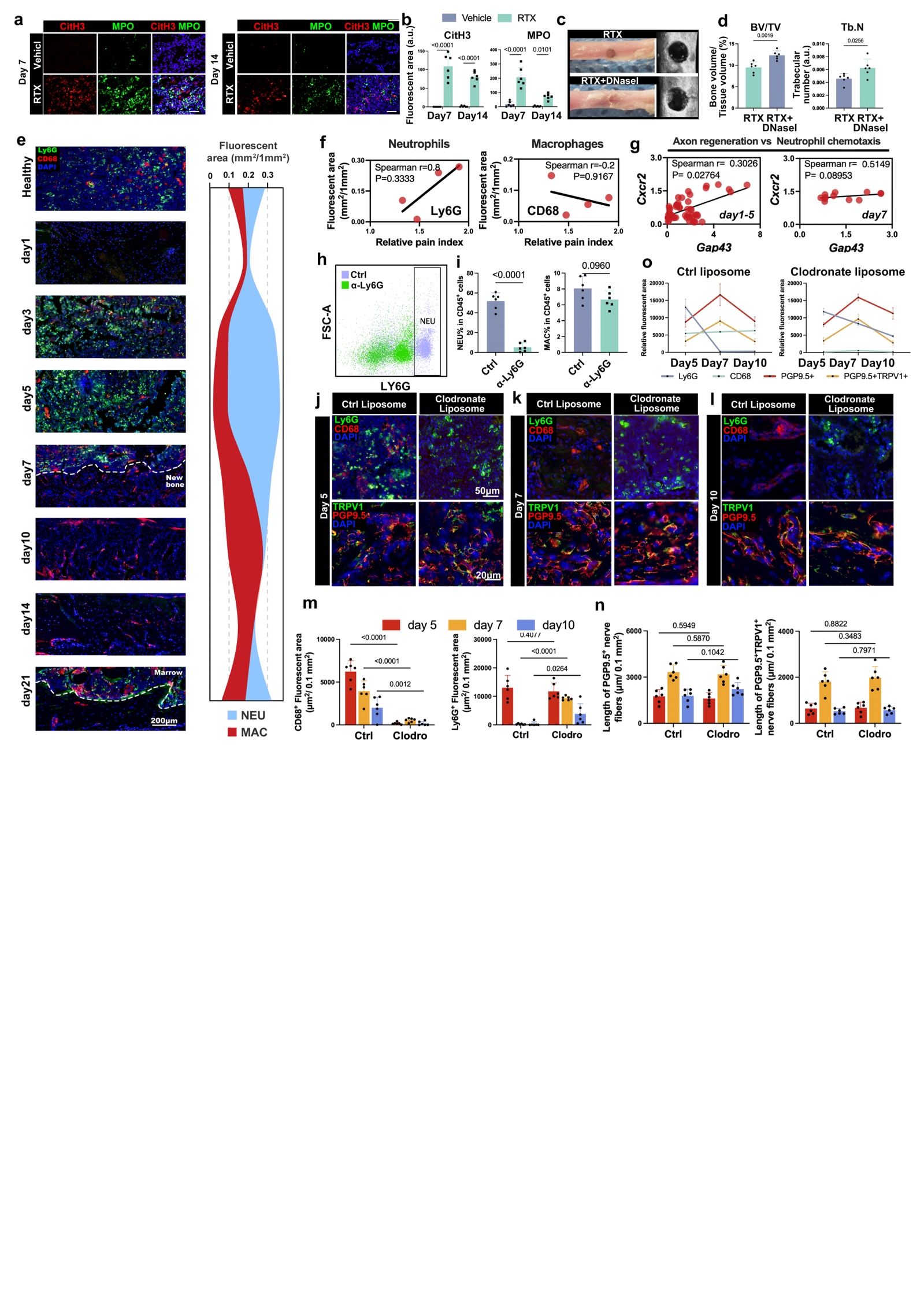
**

**Extended data Fig. 2. Neutrophils initiate nociceptive nerve sprouting**

(a) Representative images and (b) semi-quantitative analysis of MPO (green)/ CitH3 (red) immunofluorescent staining of the murine femoral diaphysis on day7 and day14 post bone injury (n = 6 femurs per group). Scale bars, 50 μm. (c) Representative images,micro-CT reconstructions and (d) quantitative analysis of BV/TV and Tb.N of the femoral defects in C57BL/6 mice (n = 6 femurs per group ). Scale bars, 1 mm. (e) Representative images of immunohistochemical staining of regenerating bone at serial time points between days 1 and 21 after injury. The stream chart presents a semi-quantitative analysis, with the width of each stream representing the fluorescent area at different time points. (f) Spearman correlation analysis between Ly6G/CD68 fluorescent area and relative pain index (n=5 time points). (g) Spearman correlation analysis among gene expression of axon regeneration marker Gap43 and neutrophil chemotaxis marker Cxcr2. (h, i) The efficiency of neutrophil depletion was verified using flow cytometry(n= 6 femurs per group). (j-l) The efficiency of macrophage depletion (up) and nociceptive ingrowth (down) was varified between day 5 and 10 (n= 6 femurs per group). (m) The semi-quantative analysis of fluorescent area of MAC (left) and NEU (right). (n) The semi-quantative analysis of the length of nerve fibers. (o) Relative length/area of MAC, NEU and nerve fibers Data are mean ± s.e.m. and are representative of at least four independent experiments. P values determined by (d, i) Student’s t test, (b, m, n) two-way ANOVA and (f, g) Spearman's rank-order correlation coefficient.


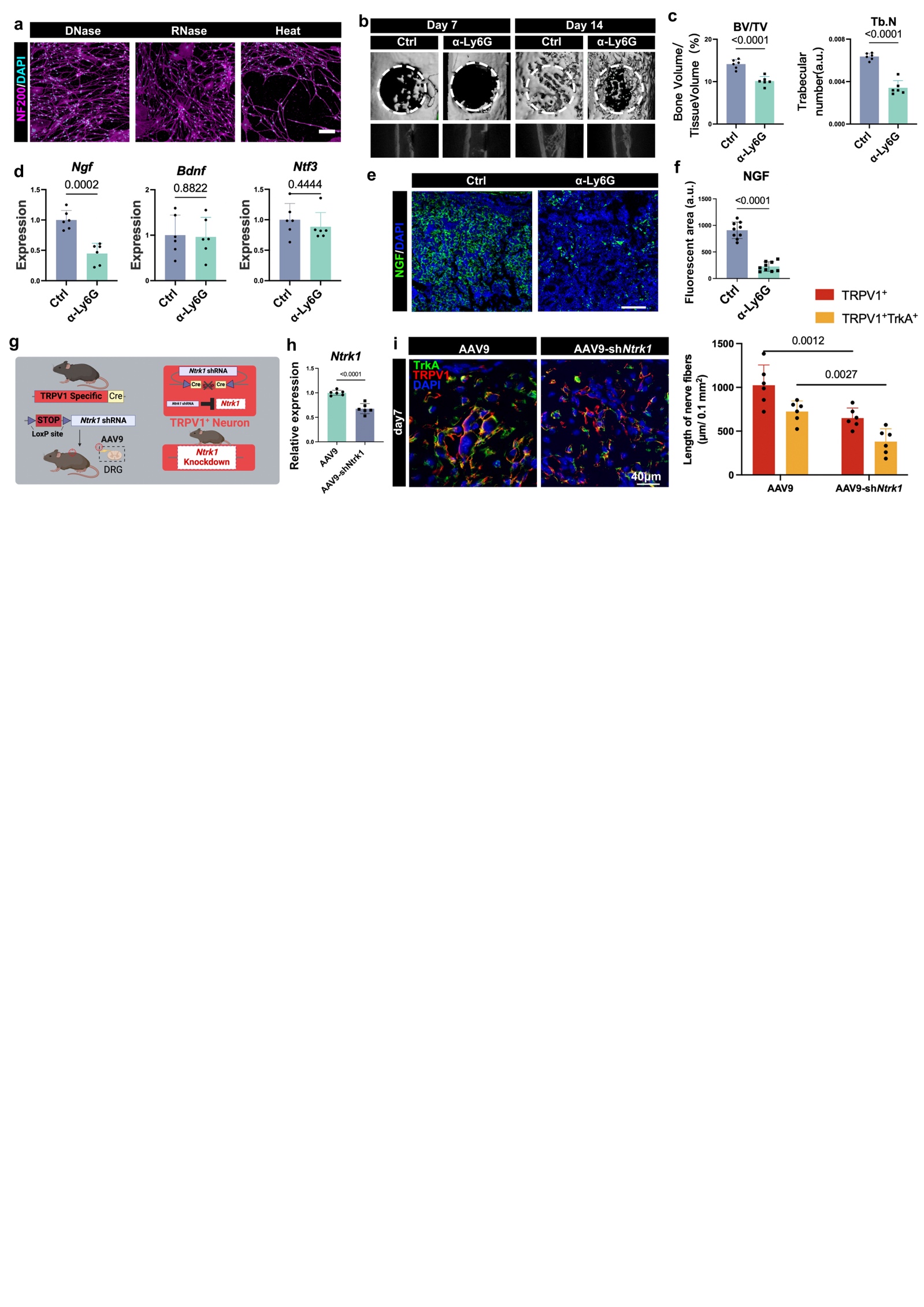


**Extended data Fig. 3. Neutrophils initiate nociceptive ingrowth by secreting NGF**

(a) Neurite outgrowth of DRG neurons triggered by NEU CM pretreated with DNase, RNAse or heat. (b) Micro-CT reconstructions and (c) quantitative analysis of bone volume/total volume (BV/TV) and trabecular number (Tb.N) of the femoral defects in indicated groups (n = 6 femurs per groups ). (d) Gene expression of neurotrophins was assessed in indicated groups (n=6 femurs per group). (e) Representative images and (f) semi-quantative analysis of immunostaining of NGF (green) in the murine femoral diaphysis on day 5. Scale bars, 100 μm. (g) Schematic diagram of conditional knockdown of Ntrk1 gene in TRPV1+ neurons. (h, i) The efficiency of conditional Ntrk1 knockdown in TRPV1+ nociceptors were verified through (h) gene expression of Ntrk1 in DRG (n=6 DRGs per group) and (i) immunofluorescent staining and quantitative analysis of the length of TRPV1+TrkA+and TRPV1+ nerve (red) in femurs isolated from TRPV1-cre mice treated with AAV9 or AAV9-shNtrk1 (n = 6 femurs per group). Scale bars, 40 μm. Data are mean ± s.e.m. and are representative of at least three independent experiments. P values determined by Student’s t-test, except for (i) two-way ANOVA.


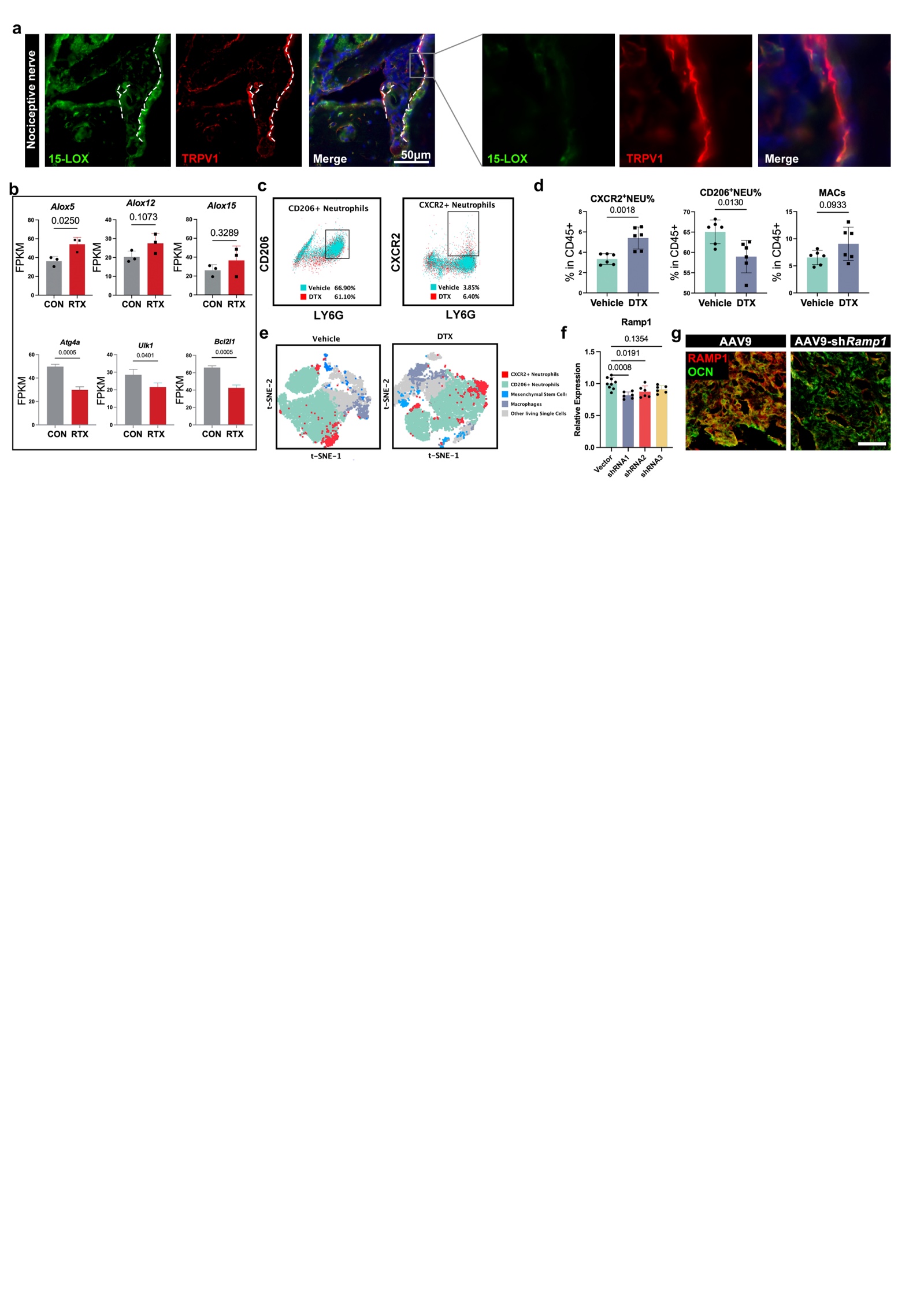


**Extended data Fig. 4. Genetic ablation of TRPV1+ nociceptive nerves induced neutrophilic inflammation and the varification of shRamp1 interference efficiency**

(a) Immunofluorescent co-localization analysis of 15-LOX and TRPV1+ nociceptive nerves, Scale bars, 50 μm. (b) The fpkm of critical gene in LXA4 biosynthesis process and autophagy (n=3). (c) Representative images, (d) percentage of NEU and MAC in CD45+ cells and (e) t-SNE analysis of flow cytometry of femoral healing tissue isolated from Vehicle- or DTX-treated TRPV1-iDTR mice on day 7 post bone injury (n=6 femurs per group). (f) The interference efficiency of three shRNA sequences was verified through RT-PCR in osteoblasts infected with lentiviral interference vector and cultured for 3 days (n=9 in control group,n=6 in experiment groups). (g) The efficiency of in vivo conditional Ramp1 knockdown in OCN+ osteoblasts was verified through immunofluorescent staining of RAMP1 (red) and OCN+ osteoblasts (green) in femur isolated from OCN-cre mice treated with AAV9 or AAV9-shRamp1. Scale bars, 50 μm. Data are mean ± s.e.m. and are representative of at least three independent experiments. P values determined by Student’s t-test, except for (f) one-way ANOVA with Tukey tests.
